# The genetic architecture of DNA replication timing in human pluripotent stem cells

**DOI:** 10.1038/s41467-021-27115-9

**Published:** 2021-11-19

**Authors:** Qiliang Ding, Matthew M. Edwards, Ning Wang, Xiang Zhu, Alexa N. Bracci, Michelle L. Hulke, Ya Hu, Yao Tong, Joyce Hsiao, Christine J. Charvet, Sulagna Ghosh, Robert E. Handsaker, Kevin Eggan, Florian T. Merkle, Jeannine Gerhardt, Dieter Egli, Andrew G. Clark, Amnon Koren

**Affiliations:** 1 Department of Molecular Biology and Genetics, Cornell University, Ithaca, NY 14853 USA; 2 Department of Pediatrics, Columbia University, New York, NY 10032 USA; 3 Department of Statistics, Pennsylvania State University, University Park, 16801 PA USA; 4 Huck Institutes of the Life Sciences, Pennsylvania State University, University Park, 16801 PA USA; 5 Department of Statistics, Stanford University, Stanford, CA 94305 USA; 6 New York Genome Center, New York, NY 10013 USA; 7 Department of Human Genetics, University of Chicago, Chicago, IL 60637 USA; 8 Stanley Center for Psychiatric Research, Broad Institute of MIT and Harvard, Cambridge, MA 02142 USA; 9 Department of Genetics, Harvard Medical School, Boston, MA 02115 USA; 10 Department of Stem Cell and Regenerative Biology, Harvard University, Cambridge, MA 02138 USA; 11 Howard Hughes Medical Institute, Harvard University, Cambridge, MA 02138 USA; 12 Wellcome Trust - Medical Research Council Institute of Metabolic Science, University of Cambridge, Cambridge, United Kingdom; 13 Ronald O. Perelman and Claudia Cohen Center for Reproductive Medicine, Weill Cornell Medicine, New York, NY 10065 USA; 14 Department of Obstetrics and Gynecology, Weill Cornell Medicine, New York, NY 10065 USA

**Keywords:** DNA replication, Epigenetics, Genome, Population genetics

## Abstract

DNA replication follows a strict spatiotemporal program that intersects with chromatin structure but has a poorly understood genetic basis. To systematically identify genetic regulators of replication timing, we exploited inter-individual variation in human pluripotent stem cells from 349 individuals. We show that the human genome’s replication program is broadly encoded in DNA and identify 1,617 *cis*-acting replication timing quantitative trait loci (rtQTLs) – sequence determinants of replication initiation. rtQTLs function individually, or in combinations of proximal and distal regulators, and are enriched at sites of histone H3 trimethylation of lysines 4, 9, and 36 together with histone hyperacetylation. H3 trimethylation marks are individually repressive yet synergistically associate with early replication. We identify pluripotency-related transcription factors and boundary elements as positive and negative regulators of replication timing, respectively. Taken together, human replication timing is controlled by a multi-layered mechanism with dozens of effectors working combinatorially and following principles analogous to transcription regulation.

## Abstract (web summary)

The genetic basis of how cells replicate their DNA is not well understood. Here, the authors identify >1000 genetic elements that control human replication and reveal a complex epigenetic system that regulates replication origin activities.

## Introduction

Eukaryotic genomes are replicated according to a strict spatiotemporal program, in which replication initiates from specific locations along chromosomes and at reproducible times. The replication timing program is a fundamental property of chromosome organization, interfaces with gene regulation, and shapes the mutational landscape of the genome. Efforts to understand the locations and nature of initiation sites and the factors that regulate DNA replication timing in mammalian cells have been ongoing for decades, with limited success^1–3^. Specifically, it is still unclear to what extent the DNA replication timing program is determined by local DNA sequences, by epigenetic factors, or by a combination thereof. Earlier studies suggested that specific sequence elements control replication initiation in human cells, with several distal and proximal elements often acting in concert^4–10^. More recently, CRISPR/Cas9-mediated deletions have suggested that several DNA sequences locally interact to control early replication in mice^11^.

Numerous lines of evidence link replication regulation to epigenetic states, in particular histone acetylations and methylations marking open chromatin^3,12–16^. However, no single epigenetic mark appears to be absolutely required nor sufficient for replication origin function. This has led to suggestions that a combination of histone marks may be required for specifying patterns of DNA replication^17^. Similarly, it has been proposed that indiscriminate DNA-binding patterns of the replication machinery may translate into a consistent, organized replication program by means of combinatorial chromatin modifications influencing subsets of replication initiation sites^3^.

The nature of such modular, combinatorial regulation of DNA replication at the genetic and epigenetic levels remains to be revealed. Previous studies applied stepwise reverse engineering approaches to probe for mechanisms controlling replication timing. However, such a complex system may be best studied with an unbiased and comprehensive interrogation of genetic and epigenetic factors and their interactions. While such an approach is currently challenging experimentally, an alternative is to take advantage of natural genetic variation. We previously showed that replication timing is variable among individuals, that it can be studied at fine-scale on a population level by sequencing the genomes of proliferating cells, and that genotype information from the same genome sequences can be used to associate replication timing variation with specific genetic polymorphisms. This results in the identification of replication timing quantitative trait loci (rtQTLs), DNA sequences that act in *cis* to affect replication initiation^18^. Leveraging human genetic variation enables the equivalent of numerous surgical genetic manipulations and their association with DNA replication timing alterations. Here, we apply this approach to hundreds of human embryonic stem cell (hESC) and induced pluripotent stem cell (iPSC) lines. Pluripotent stem cells are particularly useful for this analysis, since they are non-transformed, karyotypically stable, and highly proliferative, and have a wealth of epigenetic data available for multi-omic analyses. We identify 1,617 *cis*-rtQTLs and analyze their locations and allelic differences. These analyses delineate the architecture of human replication timing as a quantitative trait involving combinatorial regulation by several layers of epigenetic mechanisms rooted in *cis*-acting DNA sequences.

## Results

### High-resolution population-scale replication timing profiles

To comprehensively characterize human inter-individual replication timing variation and its genetic basis, we analyzed deep (~30x) whole-genome sequences of 121 hESC lines^19^ and 326 iPSC lines^20^, in addition to another 24 hESCs and 17 iPSCs (sequenced to ~16–30x) for a total of 488 cell lines (Methods). ES and iPS cultures are highly proliferative, containing 35–55% cells in S phase. DNA replication timing leads to variation in DNA copy number along chromosomes among S phase cells (e.g., early-replicating regions are duplicated in most cells), causing read depth fluctuations in the sequencing data^18^ (Supplementary Fig. 1a). Indeed, we were able to generate high-resolution replication timing profiles for a total of 140 hESCs and 317 iPSCs (Methods). ES and iPS cells had similar replication profiles, as expected.

**Fig. 1:**
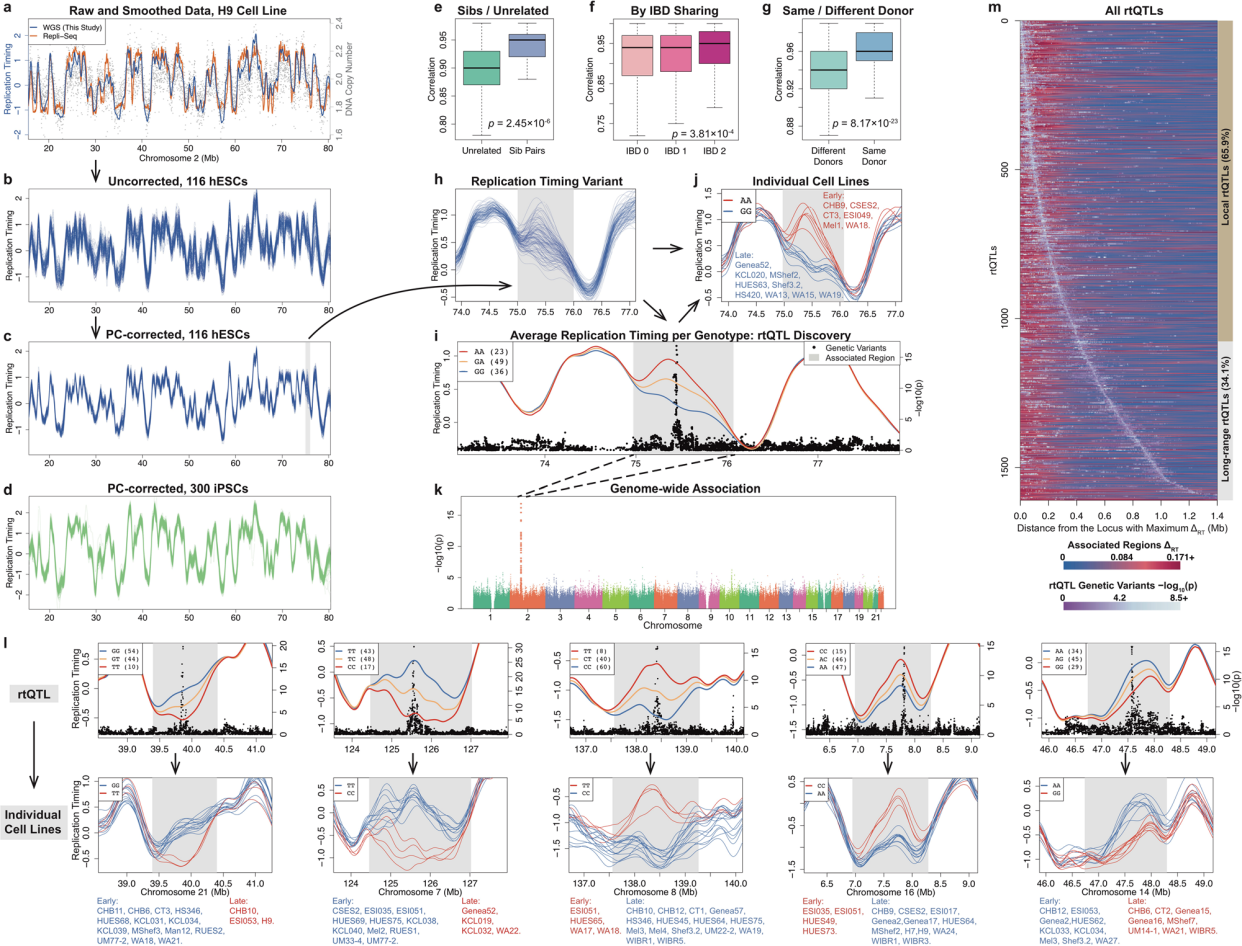
The Human Genome’s Replication Timing Program is Extensively Encoded in DNA. **a** Replication timing (blue line; *Z*-score) inferred from read depth fluctuations (gray) for the H9 cell line. Orange: Repli-Seq data for H9^74^. **b** Replication timing profiles are highly reproducible among samples. **c**, **d** PC-correction improves replication profile accuracy. **e**–**g** Genetic relatedness associated with replication timing similarity. **e** Sibling vs. unrelated hESCs (two-sided Wilcoxon rank-sum test). *n* = 5754 (pairs of genetically unrelated samples), *n* = 24 (pairs of genetic siblings). **f** Genomic regions stratified by increasing identity-by-descent (ANOVA). *n* = 14,856 (pairs of genomic regions with IBD 0), *n* = 7010 (pairs of genomic regions with IBD 1), and *n* = 262 (pairs of genomic regions with IBD 2). **g** iPSCs from the same or different donors (two-sided Wilcoxon rank-sum test). *n* = 44,742 (pairs of iPS cell lines from different donors), *n* = 108 (pairs of iPS cell lines from the same donor [derived separately]). For panels **e**, **f**, and **g**, top and bottom whiskers denote the maximum and minimum value, respectively. The top and bottom boxes denote the third and first quartile, respectively. Center denotes the median. Outliers removed using R boxplot option “outline=F”. **b** A genomic region (gray) with inter-individual replication timing variation. **i**–**k** Genetic association reveals rtQTLs. **i** A haplotype strongly associated with the replication timing variant from panel h (panel **k** genome-wide association). Mean replication timing (left *Y* axis) for individuals with different genotypes at rs12713840, the top SNP, demonstrates that SNPs in *cis* (right *Y* axis) associate with replication initiation. Gray: affected region. **j** Replication timing at the variant from panel **h**, stratifying individuals by rs12713840 genotype. Genotype is the main determinant of replication timing variation. **l** Additional rtQTL examples. **m** All rtQTLs. Each horizontal line is an rtQTL, oriented from the replication timing locus with maximum difference between genotypes (*Δ*_*RT*_), showing the averaged replication timing difference on both sides of that locus. Foreground (gray-purple) shades: rtQTL SNPs, color-coded by *p* values, and placed according to their distance to the locus of maximal *Δ*_*RT*_. Most rtQTLs influence surrounding genomic region (“local”), while a subset show long-range effects.

Replication timing profiles were continuous along chromosomes, highly reproducible among samples (median *r* = 0.93), and consistent with previous replication timing measurements by Repli-Seq (median *r* = 0.86; Fig. 1a–d). The replication profiles were exceptionally sharp, in line with recent high-resolution Repli-Seq data^21^, with discrete peaks and valleys (local maxima and minima) that were themselves highly reproducible among individuals. Replication timing peaks represent prominent initiation sites containing one or more replication origins. We further improved data resolution using principal component (PC)-based correction across cell lines (Fig. 1c and d, Supplementary Fig. 1b; Methods), resulting in a median correlation *r* = 0.98 among hESCs, *r* = 0.97 among iPSCs and *r* = 0.85 between the two cell types.

### DNA replication timing is broadly influenced by *cis*-acting sequences

While replication timing profiles were highly reproducible among individuals, we nonetheless observed genomic regions with substantial inter-individual variation. We identified 1,489 autosomal replication timing variants in hESCs and 1837 in iPSCs, cumulatively encompassing 795 Mb (34%) and 980 Mb (40.8%), respectively, of the analyzable genome (Fig. 1c, d). We hypothesized that at least some of this variation is due to genetic polymorphism. To test this, we first compared replication timing variation between 24 pairs of hESC lines that are genetic siblings, versus unrelated cell lines; between genomic regions that are identical by descent (IBD), half-identical or non-identical between sibling cell lines; and between 108 pairs of iPSC lines derived from the same donor, compared to different donors (Methods). Consistent with a significant genetic contribution to replication timing variation, samples or genomic intervals that are genetically related consistently showed greater replication timing similarity than unrelated comparisons (Fig. 1e–g). Using pairs of iPSC lines derived from the same donor, we observed substantial and consistent donor effects across replication timing loci genome-wide (median: 20.7%, maximum: 71.9% of the total variance). Donor effects accounted for the largest proportion of variance in 33.1% of replication timing loci. These results were comparable with previous observations of donor effects on iPSC gene expression^20^, and suggest that differences between donors substantially impact iPSC replication timing.

To further dissect genetic contributions to replication timing variation, we used our previously described rtQTL mapping approach^18^ to associate replication timing with specific genetic polymorphisms. We limited this analysis to 108 hESCs of European ancestry and to 192 iPSCs from different individuals.

We identified 1617 *cis*-rtQTLs (FDR 0.1; 1,012 were identified with FDR 0.05; Fig. 1i–m; Supplementary Data 1), two orders of magnitude more than previous associations of replication timing with *cis*-acting sequences^11,18^. The greater success in rtQTL identification was mainly driven by the more deeply sequenced genomes, the high proliferation rate of stem cells and by the improved analytical framework (Methods, Supplementary Fig. 1c). We used CAVIAR^22^ to fine-map (90% credible level; Supplementary Data 2) a median of 33 SNPs per rtQTL, with 316 rtQTLs mapped to within 10 SNPs and 36 rtQTLs mapped to no more than three SNPs. rtQTL mapping was cross-validated between ES and iPS cells and further confirmed using additionally-sequenced cell lines and with a locus-specific single-molecule assay (Supplementary Fig. 2). We found that replication timing loci that harbor rtQTLs had significantly greater donor effects than other loci (median: 20.8% vs. 18.8%, Wilcoxon rank-sum *p* << 2.2×10^-16^). We further explicitly modelled rtQTL genotype in variance component analyses of replication timing at the loci with the strongest association of each iPSC rtQTL, and found that on average, 54.7% of donor effects (17.9% of the total variance) can be explained by rtQTL genotype. These findings indicate that genetic differences substantially contribute to the donor effects on replication timing.

rtQTLs influenced the replication timing of regions spanning 858 kb on average and a total of 741.8 Mb of genomic sequence (31.8% of the genome, Fig. 1m). This is a lower bound estimate of the extent to which human replication timing is influenced by DNA sequence, since our approach will not detect weaker rtQTLs, invariant sequences or rare variant rtQTLs. Intriguingly, 67.9% (1,098) of rtQTLs coincided with sharp peaks in the replication profiles (binomial *p* = 2.24×10^-25^; Fig. 1i–l), and rtQTL SNPs were significantly closer to peaks than expected (Wilcoxon rank-sum *p* = 1.77 × 10^-16^). This suggests that rtQTLs may influence replication initiation, as previously reported^18,23^, and that most rtQTLs can be used as fine-scale markers of replication initiation regions. We ruled out that rtQTLs are predominantly markers of gene expression variation: only 13.7% of iPSC rtQTLs were associated with variation in expression of genes overlapping rtQTL-affected regions (14.6% when extending an additional 1 Mb on each side). The identification of rtQTLs as precise genetic determinants of replication timing provides a unique opportunity to fine-map molecular mechanisms controlling replication initiation and timing.

### A promoter-enhancer logic of replication timing regulation

**Fig. 2:**
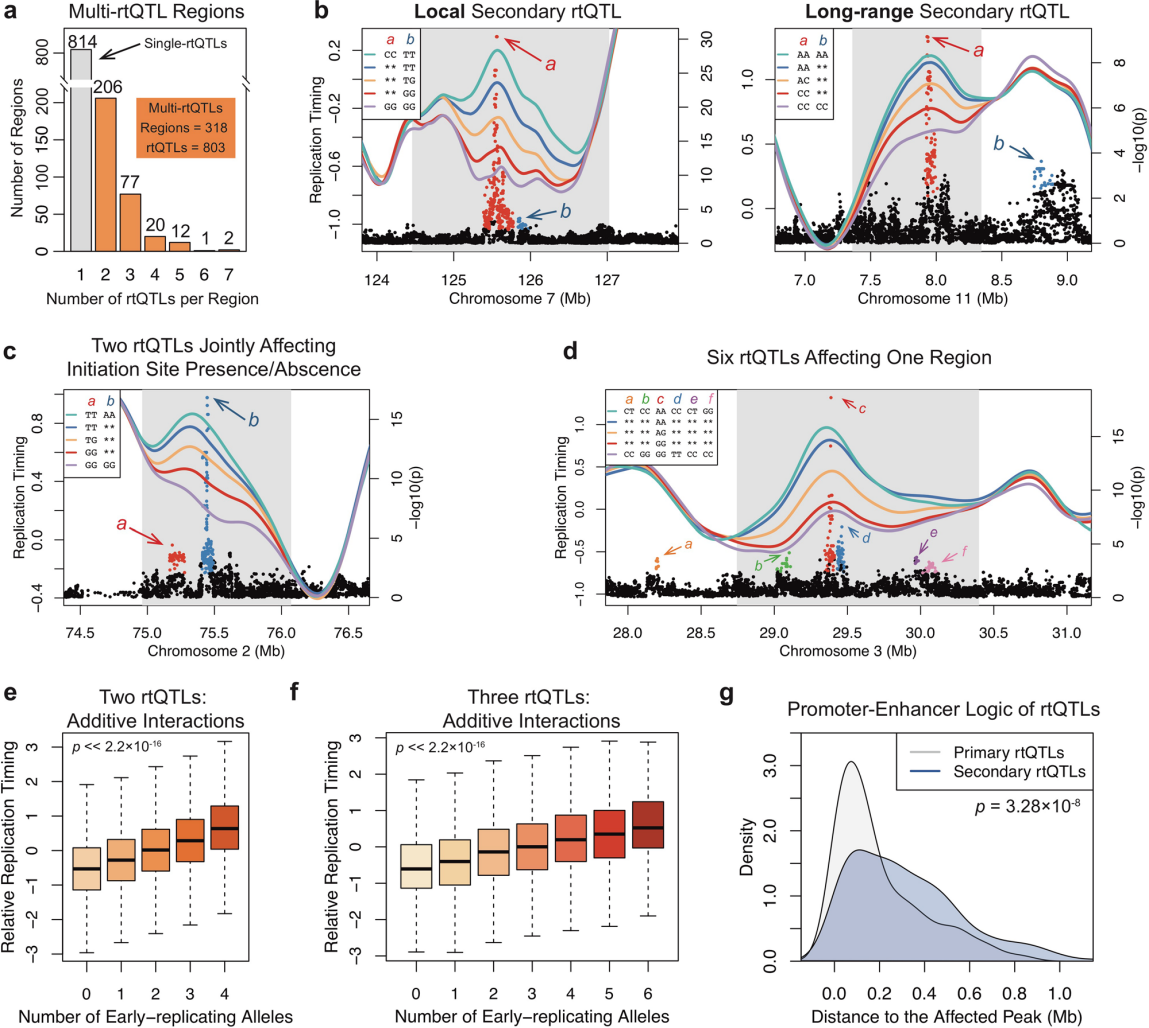
Multiple DNA Sequences Interact to Regulate Replication Timing. **a** Hundreds of regions are controlled by multi-rtQTLs. **b**, **c** Two rtQTLs affecting the same region. Blue, yellow, and red lines represent one rtQTL. Purple and green lines represent the mean replication timing of individuals carrying the late- or early-replicating genotypes, respectively, at both rtQTLs. Considering both rtQTLs explains a larger fraction of variation (green lines are higher than blue lines; conversely for purple/red lines). Asterisks (in legends): any genotype at this rtQTL. In panel c, the GG/GG combination of alleles is associated with complete loss of initiation activity. **d** A replication initiation site associated with six rtQTLs. Each rtQTL was significant even after conditioning on all other five rtQTLs in the region. **e**, **f** rtQTLs exert additive effects. All regions with two (**e**) or three (**f**) rtQTLs were pooled; replication timing is linearly correlated to the number of early-replicating alleles (*p* values based on linear regression). **e**: *n* = 5416, 15,820, 23,551, 16,181, and 7158 genotype-replication timing pairs (for 0–4 early-replicating alleles, respectively). f: n = 770, 2136, 3844, 4310, 3520, 2039, and 661 genotype-replicating timing pairs (for 0–6 early-replicating alleles, respectively). For panels **e** and **f**, the top and bottom whiskers denote the maximum and minimum value, respectively. The top and bottom boxes denote the third and first quartile, respectively. The center denotes the median. Outliers were determined and removed using R boxplot option “outline=F”. **g** Multi-rtQTLs conform to a “promoter-enhancer” logic, primary rtQTLs being closer to the affected replication timing peak than secondary rtQTLs (two-sided Wilcoxon rank-sum test p-value indicated).

We first used rtQTLs to examine the *cis*-regulatory logic of human replication timing. We observed that a subset of rtQTLs are distal to their associated genomic region (Fig. 1m), and that in many regions, separate rtQTLs clustered in close proximity. This suggests that multiple DNA sequences, local and distal, could interact to affect the replication timing of a given locus. We identified 318 cases, encompassing 803 rtQTLs, where at least two, and strikingly, up to seven rtQTLs were associated with the same region, each providing additional explanatory power (Fig. 2a–d). We call these “multi-rtQTL” regions and refer to the strongest rtQTL as the “primary”, while all other rtQTLs are “secondary”. In some cases, one rtQTL quantitatively influenced replication timing, while several rtQTLs together explained the actual presence of active initiation (Fig. 2c). Thus, replication initiation is regulated along a continuum, one extreme of which is no activity at all despite the presence of a potential initiation site.

A recent study^11^ suggested that synergistic interactions between *cis*-acting elements are required for controlling early replication. We directly tested for interactions between primary and secondary rtQTLs at regions that harbored two rtQTLs, hence between zero and four early-replicating alleles. We further pooled all genomic regions containing three or four rtQTLs and evaluated the relationship between the number of early-replicating alleles and the replication timing of the associated regions. Replication timing showed a linear relationship with the number of early-replicating alleles (linear regression *p* << 2.2×10^-16^; Fig. 2e, f), and none of the individual regions showed evidence for synergistic interactions between rtQTLs. We also systematically searched for interactions between pairs of genetic variants in affecting replication timing, yet identified none (Methods). While these findings do not rule out synergistic relationships, they suggest that rtQTLs operate in a predominantly additive manner.

Of the 318 multi-rtQTL regions, 176 were associated with replication timing peaks. In 115 of these cases (65.3%), primary rtQTLs were closer to the peak than secondary rtQTLs (Fig. 2g, *p* = 3.28×10^-8^). This resembles eQTLs (expression QTLs), in which primary eQTLs show stronger enrichment at promoters, while weaker eQTLs are enriched at enhancers^24^. Also in resemblance to enhancers and promoters, primary and secondary rtQTLs tended to cluster in nuclear space (based on Hi-C data) more than expected by chance (*p* = 9.73×10^-3^, *Z* test, Supplementary Fig. 3). Drawing from this analogy, we propose that rtQTLs may follow a logic akin to promoters and enhancers, in which primary rtQTLs function as main *cis*-acting regulators of replication initiation, while other sequences, marked by secondary rtQTLs, serve as distal regulatory elements that fine-tune the replication dynamics of a given region.

### Histone modifications associated with DNA replication initiation

We next utilized the basepair-resolution sequence-specificity of rtQTLs to investigate the molecular mechanisms of DNA replication timing. We initially considered rtQTL locations per se, independently of allelic variation. Since extensive epigenetic data were available for seven of the hESC lines in our dataset, we focused this analysis on hESCs and used iPSCs for validation. Given that replication initiation in mammalian cells is thought to occur in diffuse regions encompassing at least several kbs, we initially considered inclusive chromatin data (“gappedPeak”), combined from several cell lines when possible, and subsequently validated our findings in individual cell lines (Methods). Consistent with previously described correlations between early replication and open chromatin^3^, rtQTLs were enriched for active chromHMM states including enhancers and transcription start sites (although they were not specifically associated with genes; Supplementary Fig. 4), DNase I hypersensitivity sites (*p* = 2.62×10^-8^, 4.11×10^-19^ in iPSCs), and H2A.Z sites^25^ (*p* = 6.69×10^-4^; *p* = 3.20×10^-17^ in iPSCs). rtQTLs also significantly overlapped with 24 histone marks (25 in iPSCs), of which 20 were active marks (Supplementary Fig. 4). The majority of these histone marks were acetylations, including several not previously linked to replication timing, for example, H2BK120ac, H2BK12ac, and H2BK20ac. H3T11ph was also consistently enriched at hESC and iPSC rtQTL sites, and so were, modestly, methylated forms of H3K4. No significant enrichments were found when the analysis was repeated on genomic loci that were distant from rtQTLs or replication timing peaks, but that matched their timing distribution (Supplementary Fig. 1i and j). This suggests that replication timing per se is unlikely to explain the epigenetic marks associated with rtQTLs.

**Fig. 3:**
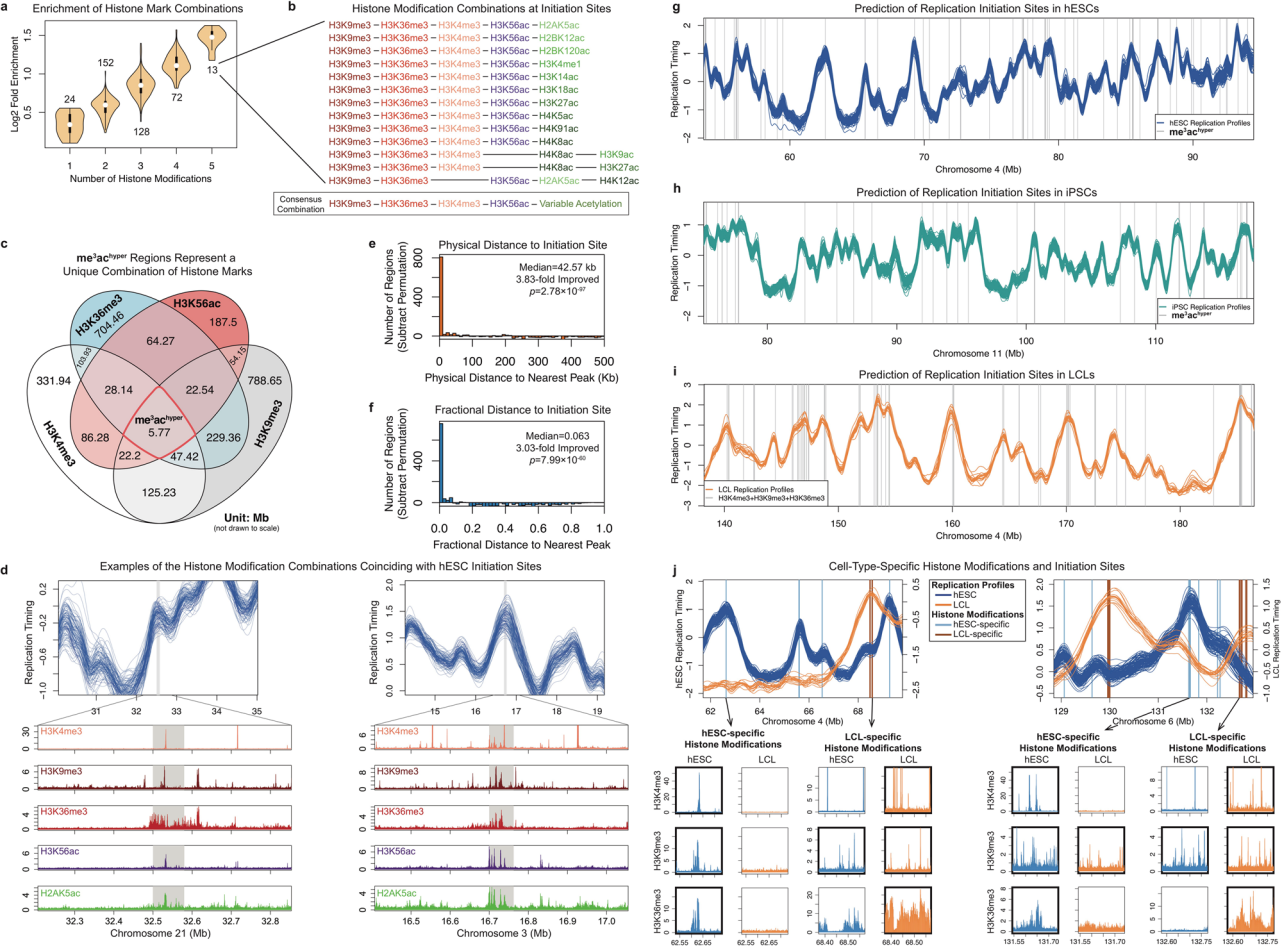
Histone Modifications Associated with Replication Initiation. **a** Iterative identification of histone mark combinations enriched at rtQTLs. Shown are enrichment distributions; the number of combinations in each category is indicated. Fold-enrichment increases gradually and is maximal for five-mark combinations. n indicated in the plot, unit: histone mark combinations. **b** Histone modification combinations for human replication initiation. The 13 combinations of five histone marks converged to a consensus combination. See Supplementary Table 1 for additional details. **c** The histone modifications (independent of rtQTLs) represents a rare combination of both active and repressive histone marks. me^3^ac^hyper^ regions comprised 0.7–3% of the regions that carry the individual histone marks. **d** Examples of histone mark combinations (Roadmap Epigenomics imputation)^72,73^ coinciding with replication timing peaks not identified as rtQTLs. **e**, **f** Distribution (after subtraction of permutations) of physical (**e**) and fractional distances (**f**) of the me^3^ac^hyper^ locations to the nearest replication timing peak. We also indicate the fold-improvement in median distance to the nearest peak compared with random expectation. See Supplementary Fig. 5c, d for further comparison with random. Panels **e** and **f** used a one-sided Wilcoxon rank-sum test. Correction for multiple testing was not applicable, as there was only one test for each sub-plot. **g** Combination of histone marks (gray, me^3^ac^hyper^ locations) predict replication initiation sites in hESCs. **h**, **i** Histone modification locations (gray vertical lines) correspond to replication timing peaks in iPSCs (**h**) and LCLs (i). **j** Cell-type-specific histone modification locations mark cell-type-specific replication initiation sites. At regions with distinct replication timing profiles for hESCs and LCLs, LCL (hESC)-specific replication timing peaks are predicted by LCL (hESC)-specific histone modification locations. Lower panels: initiation sites coincide (thick borders) with all three histone trimethylation marks in the cell type in which they are active, but with one or none of the marks in the cell type in which they are inactive.

Of note, the histone mark enrichments were modest, with each present at between 165 to 541 (median: 429) of 608 hESC rtQTLs (median: 542 of 1167 iPSC rtQTLs), while each rtQTL overlapped 20 histone marks on average. We surmised that this abundance of histone modifications may be suggestive of combinatorial regulation. To test this, we systematically searched for combinations of histone marks with stronger enrichments at rtQTLs when considered jointly (Methods). We identified 152 combinations of two overlapping histone marks that were more enriched than the individual marks. We further identified 128 co-enriched three-mark combinations, 72 four-mark combinations, and 13 five-mark combinations (enrichment *p* values: 2.42×10^-37^–1.09×10^-45^), at which point no further improvements in enrichment were obtained (Fig. 3a; Supplementary Data 3). Although these enrichments were relatively modest, finding higher-order combinations of histone modifications at rtQTL sites suggested a potentially novel principle of replication timing regulation. Importantly, since these enrichments controlled for replication timing, they were not identified because they mark early replicating regions, but because they specifically mark rtQTL locations, and, by inference, replication initiation sites. Repeating this analysis using early vs. late-replicating peaks, or using peaks vs. valleys, only revealed individually enriched histone marks or pairs thereof, but not higher order combinations. Thus, our ability to identify the myriad epigenetic factors and combinations related to replication timing is directly related to our approach of rtQTL mapping in a population cohort.

Strikingly, all 13 combinations of five histone marks contained the trimethylation marks H3K9me3 and H3K36me3, and 12 of the combinations also contained H3K4me3. In addition, all 13 combinations included at least one histone acetylation mark. H3K56ac was included in 11 of the combinations, while the additional acetylations occurred on variable histone residues (Fig. 3b). The appearance of H3K56ac in multiple combinations was attributed to its more unique presence across the genome compared to the other histone acetylations, which tended to be redundant with each other (Supplementary Fig. 5a, b). Indeed, various acetylation marks often coincided with the five histone mark combinations, e.g., in 70.8% of the cases, 11 or more acetylation marks co-occurred at the location of a five-mark combination. We term this combination of three H3 trimethylations together with hyperacetylation the “me^3^ac^hyper^ histone modifications”. Both the enrichment of individual histone modifications and the me^3^ac^hyper^ combinations were also observed in individual cell lines, the latter when extending the overlapping regions by 1 kb on each side of the modification peaks (Figs. S6 and S7). Thus, these modifications occurring in close proximity to each other may mark the locations of replication initiation sites. We note, however, that our data does not necessarily imply that all these modifications fall at the exact same genomic sites or on the same nucleosomes. Genome-wide, there were 6,670 such locations in hESCs (Supplementary Data 4) and a total of 5120 identified in individual cell lines (median of 633 per cell line). They covered a median of 635 bp (1597 bp with the extended overlap requirement) and cumulatively encompassed 0.24% (0.31%, respectively) of the genome, thus they represent specific, relatively localized genomic sites.

Importantly, when considered individually, the implicated histone modifications only showed weak enrichments (Supplementary Figure 4c). H3K9me3 and H3K36me3, in particular, showed marginal or no enrichment at rtQTLs. H3K9me3 is a marker of heterochromatin (although has been observed in the bodies of actively transcribed genes)^26^, while H3K4me3 marks gene promoters and H3K36me3 is typically present in gene bodies. These histone trimethylations are largely mutually exclusive. However, in rare cases, they coincide in nearby genomic locations (Fig. 3c, d). These trimethylation marks with disparate effects on chromatin structure, together with histone hyperacetylation, comprise a unique chromatin state that is distinct from previously described bivalent chromatin (e.g., the combination of H3K4me3 and H3K27me3)^27^. It is in these rare locations of unusual chromatin structure that rtQTLs tend to be present.

The me^3^ac^hyper^ histone modifications appeared to be specific to DNA replication initiation rather than to transcription, as only 5.2% of histone modification regions overlapped with the transcription start site (TSS) of an active gene and the majority (71.2%) of rtQTLs that overlapped the histone modifications did not associate with gene expression variation. On the other hand, G-quadruplexes were enriched at me^3^ac^hyper^ histone modification sites (Supplementary Figure 4k–n), consistent with previous reports of replication origin mapping^28–31^.

Subsets of the identified histone mark combinations have been previously linked to the recruitment of components of the replication machinery to specific chromosomal locations. Histone H3 trimethylation of lysines 4, 9, and 36 have been shown to exert a cross-talk that serves as an “epigenetic addressing system” for site-specific replication initiation^32,33^. They recruit KDM4 and KDM5 family histone demethylases, which directly interact with, and/or are required for recruitment to DNA of MCM, PCNA, DNA polymerases, and other replication factors^32–36^. H3K4me3 also synergizes with flanking H3K9ac and H3K14ac (both identified as part of the me^3^ac^hyper^ histone modifications) to recruit chromatin readers to DNA^37^. Another study showed that histone hyperacetylation synergizes with H3K9me3 to promote early replication of otherwise late-replicating mouse chromocenters^38^. In turn, acetylated histones have been shown to recruit replication initiation factors including TICRR/TRESLIN, ORC, and MCM, via mediators such as BRD2, BRD4, and the histone acetyltransferase HBO1 (histone acetyltransferase binding to ORC)^13,39–41^. In particular, HBO1 promotes MCM loading by acetylating H4 on lysines 5, 8, and 12, and subsequently promotes origin activation by acetylating H3K14^42,43^; we identified all of these acetylations as part of the me^3^ac^hyper^ combinations. Moreover, H4K12ac, the most strongly enriched mark at rtQTLs, is a preferred target of HBO1 at replication origins^39,41^. These biochemical evidence provide a plausible explanation for the combination of histone marks being associated with replication initiation activity.

Taken together, we identified a combination of histone marks, consisting of three trimethylated H3 residues (H3K4me3, H3K9me3, H3K36me3) together with H3K56ac and broadly hyperacetylated chromatin that consistently coincide with rtQTLs. To further test the involvement of these histone modifications in replication initiation, we analyze below their association with: (1) replication timing peaks in general (independent of rtQTLs); (2) replication timing peaks in other cell types; (3) replication timing peaks that vary between cell types; and (4) replication timing variation among individuals at rtQTLs.

### Combinations of histone modifications predict replication initiation sites across cell types

We considered whether the histone modification combinations could be a general property of replication initiation sites, revealed by leveraging the base-pair resolution of rtQTLs, but not limited to rtQTLs. We therefore tested whether the histone modifications also associated with the larger number of replication timing peaks (found in >10% of the samples; Supplementary Data 5) not identified as rtQTLs (81.5% of all peaks). While the probabilities of having a peak near the 24 individually enriched histone marks were significantly greater than expected (one-tailed Wilcoxon rank-sum *p* = 4.96×10^-17^) and were greatest at the actual histone mark sites (Supplementary Fig. 5e–g), individual histone marks are very common in the genome and insufficient for predicting peaks. We refined prediction through combining histone marks (Supplementary Fig. 5h and i), demonstrating a gradual increase in association with peaks up to the five-mark combinations, which were significantly more likely than expected to coincide with peaks (Supplementary Fig. 5e–g, *p* = 4.10×10^-10^). me^3^ac^hyper^ sites had an even higher likelihood of overlapping peaks (Supplementary Fig. 5e–g). Consistently, the distances of me^3^ac^hyper^ regions to the nearest peak were substantially shorter than random permutations (42.57 kb; fold-improvement over random: 3.83-fold [physical], 3.17-fold [fractional]; Fig. 3e, f; Similar results were obtained for the histone modification combinations verified in individual cell lines: median distance to nearest peak of 42.10 kb). For example, 41.7% of the me^3^ac^hyper^ sites corresponded to replication timing peaks within 10 kb (positive predictive value; *Z*-test *p* << 2.2×10^-16^, 1.70-fold enriched over random). Conversely, 60.3% of peaks were located within 10 kb of predicted regions (sensitivity; *p* << 2.2×10^-16^, 1.51-fold enriched). We further evaluated the prediction performance of the me^3^ac^hyper^ regions visually (Fig. 3g) and with receiver operating characteristic (ROC) curves (Supplementary Fig. 5j–l, area under the ROC curve [AUC_ROC_] for me^3^ac^hyper^ regions: 0.776). Peaks predicted by histone marks replicated earlier than other peaks (median: 0.61 vs. 0.14, Wilcoxon rank-sum *p* = 6.58×10^-53^) and were locally more prominent (timing difference compared to flanking valleys, median: 0.32 vs. 0.18, *p* = 1.19×10^-17^). Consistently, the replication profiles surrounding me^3^ac^hyper^ sites formed a sharp peak (Supplementary Fig. 5o).

The histone modification combinations were substantially more specific than DNase I hypersensitivity (Supplementary Fig. 5q), which was previously suggested to explain 87% of replication timing profiles^44^. In addition, the ability to predict the locations of replication initiation sites was even greater for local clusters of the histone modifications. We found that genomic regions containing multiple nearby instances of the me^3^ac^hyper^ histone modifications, and/or histone modification instances spanning longer regions, were closer to replication timing peaks than sparser occurrences of the modifications (Supplementary Fig. 5m). For example, local regions (10 kb windows) having at least 30% covered by the histone modifications had a median distance of 4.93 kb to peaks, an 8.75-fold improvement compared to all me^3^ac^hyper^ histone modification regions and an 32.8-fold improvement compared to random. This is consistent with the idea that chromatin structure influences DNA replication initiation in a regional manner, and that clusters of these histone modifications function cooperatively to promote DNA replication initiation.

In order to test whether the histone modification combinations could be a consequence of early replication rather than a potential cause of replication initiation, we tested genomic loci with matched replication timing (difference < 0.5) that were distant (>500 kb away) from the center of replication timing peaks. The median distance from histone modification sites to these loci was 843.25 kb, compared to 43.16 kb for replication timing peaks (19.54-fold different) (Supplementary Fig. 5n). Thus, the histone modification combinations associate specifically with replication initiation.

Taken together, the combinations of nearby histone marks that are enriched at rtQTLs predict ~60% of initiation site locations across the genome at 10 kb resolution, even for those sites without rtQTLs, and particularly for the early and most prominent initiation sites. These histone mark combinations may thus promote replication initiation not just at specific genomic loci, as previously proposed^32,33,45^, but across a large fraction of the genome. We note, though, that some replication timing peaks did not co-localize with histone modification locations, thus there must be additional mechanisms independently specifying replication initiation sites, underscoring the complexity of mammalian replication initiation.

An even more rigorous test of the five-mark combinations being indicators of replication initiation is whether they could predict the location of replication timing peaks in other cell types. Examining the histone modifications (identified in hESCs) in both iPSCs (Supplementary Data 6) and lymphoblastoid cell lines (LCLs; for which we only had experimentally measured data for the trimethylation marks)^46–48^, we found that the same histone modifications can predict initiation sites (Supplementary Data 7 for iPSCs) as accurately and specifically as in hESC (Fig. 3h, i), and similarly associates with early replication (Supplementary Figure 5o). In particular, LCLs have epigenetic and replication timing landscapes that are distinct from those of hESC (and iPSCs). In genomic regions at which LCL and hESC replication timing differed, LCL-specific histone modification locations corresponded to LCL-specific initiation sites, and vice versa for hESCs (Fig. 3j). Predicted cell-type-specific initiation sites resided in early-replicating genomic regions in the corresponding cell type, but not in other cell types (Supplementary Fig. 5p). Incorporating imputed (computationally predicted) LCL acetylation data further improved prediction performance from a median distance of 177.49 kb to the nearest replication timing peak for me^3^, to 130.69 kb for me^3^ + imputed H3K56ac, and down to 119.01 kb for me^3^ + imputed ac^hyper^. Thus, the histone modification combinations characterizes and predicts cell-type-specific replication initiation.

### Co-variation of replication timing and histone modifications reveals combinatorial control of replication timing

The previous analyses considered rtQTL locations per se. However, since rtQTLs represent replication timing variation among individuals, their allelic differences provide a powerful opportunity to investigate molecular mechanisms controlling replication timing. In particular, given that specific histone marks associate with replication initiation, we predicted that rtQTL SNP alleles will be associated with variation in the abundance of these marks among individuals.

**Fig. 4:**
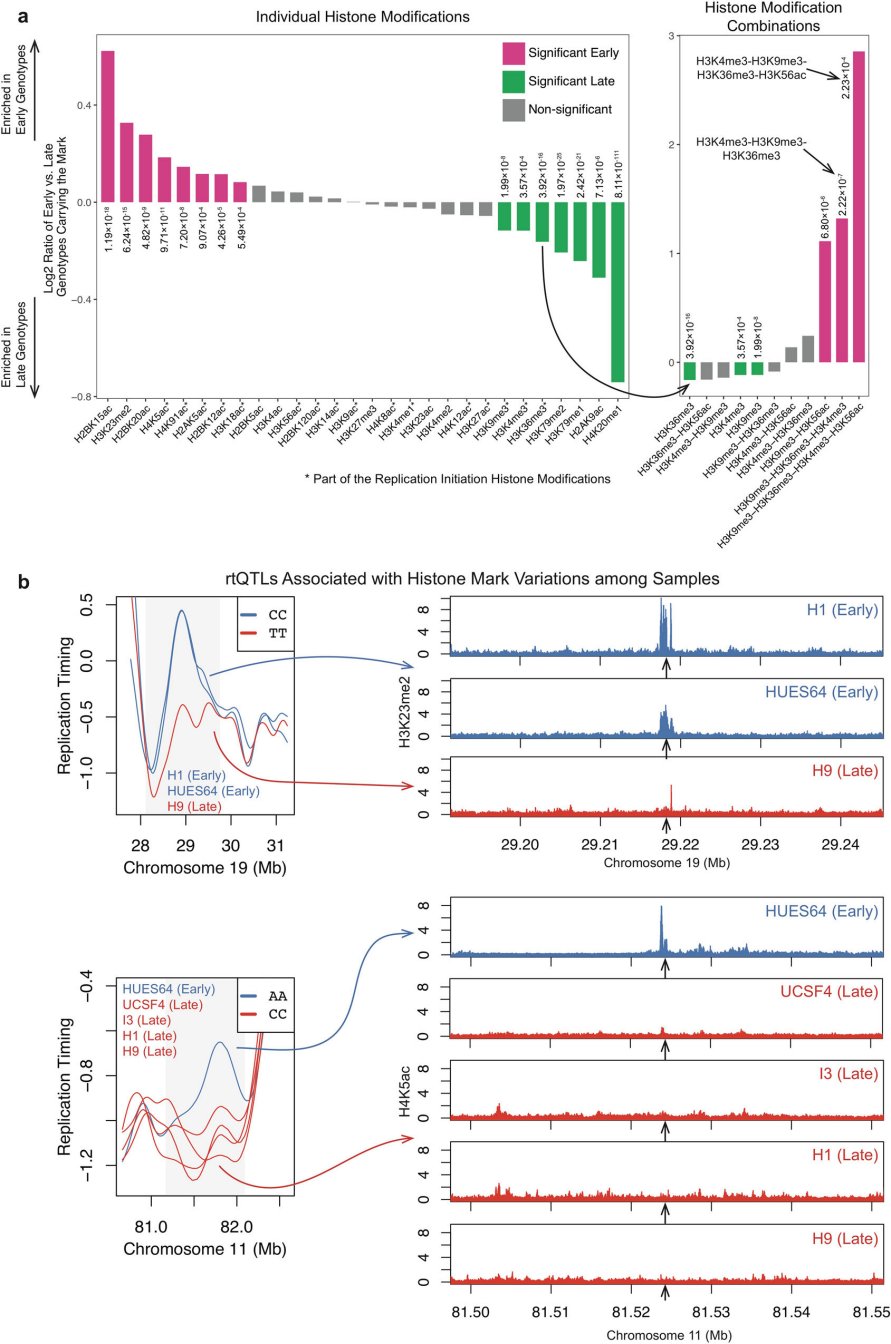
Histone marks associate with DNA replication timing. **a** Association of rtQTL genotypes with individual (left panel) or combinations (right panel) of histone marks. Positive (negative) values indicate that individuals with early (late)-replicating genotypes are more likely to carry a histone mark at those rtQTL sites. Right panel: while individual H3 methylation marks associate with late replication, the H3K4me3-H3K9me3-H3K36me3 combination is strongly associated with early replication, and even more so when combined with H3K56ac. Note the different Y scale. **b** Examples of rtQTLs associated with histone mark variations. Replication timing and corresponding histone ChIP-seq tracks for individual cell lines homozygous for the early- or late-replicating alleles. Early replication correlates with the presence of the specified histone marks.

We took an unbiased approach using seven hESC lines with both replication timing and histone modification data (Methods). Cell lines carrying early replicating genotypes at rtQTLs were more likely than individuals with late-replicating genotypes to harbor active histone marks and chromHMM states at those rtQTL sites (Fig. 4 and S8). Across individuals and genomic sites, eight histone modifications were consistently present in individuals with rtQTL alleles indicative of early replication. Of those, seven were acetylations, consistent with histone acetylation promoting early replication^3,12–16,39,41^. Of the 12 acetylation marks that are part of the replication initiation histone modification combinations, nine individually associated with early replicating rtQTL genotypes (five of which reached statistical significance). We also identified seven modifications that consistently coincided with late replicating alleles, of which six were methylation marks (Fig. 4a); Thus, histone methylation emerges as being generally repressive for replication.

Counter-intuitively, the histone trimethylation marks (H3K4me3, H3K9me3, and H3K36me3) were individually more likely to be associated with late-replicating genotypes (Fig. 4a). In contrast, the combination of all three trimethylation marks was 2.5-times more likely to be carried by early-replicating than by late-replicating genotypes. Furthermore, a combination that also included H3K56ac was 7.24-times more likely to be carried by early-replicating genotypes (Fig. 4a). Thus, these marks appear to individually act as weak repressors of replication but act synergistically, in noncanonical ways, to strongly promote early replication. Taken together, the involvement of me^3^ac^hyper^ in replication initiation is supported by several lines of evidence: enrichment at rtQTLs (Fig. 3a); correspondence with replication timing peaks in general, and across several cell types (Fig. 3d–i, Supplementary Figure 5e–g, j, and o); co-variation with cell-type-specific replication initiation patterns (Fig. 3j and Supplementary Fig. 5p); and correlation with inter-individual replication timing variation (Fig. 4).

### DNA-binding factors modulate DNA replication timing

The above results indicate that *cis*-acting sequences, manifesting as rtQTLs, influence the positions and timing of replication initiation by associating with histone modifications. To identify additional factors that influence replication timing via *cis*-acting sequences, we analyzed the binding sites of 51 DNA binding factors in hESCs^46,49^. The binding of eight factors was significantly enriched at rtQTLs, including the main pluripotency factors SOX2, POU5F1 (OCT4), and NANOG, the latter two reproducible with available data in iPSCs (Supplementary Fig. 9). Three chromatin remodelers, EP300 (P300), SP1, and RBBP5, were also enriched at rtQTLs. EP300 is a histone acetyltransferase that catalyzes at least six acetylation marks in the replication initiation histone modification combinations, including H3K56ac^50^.

**Fig. 5:**
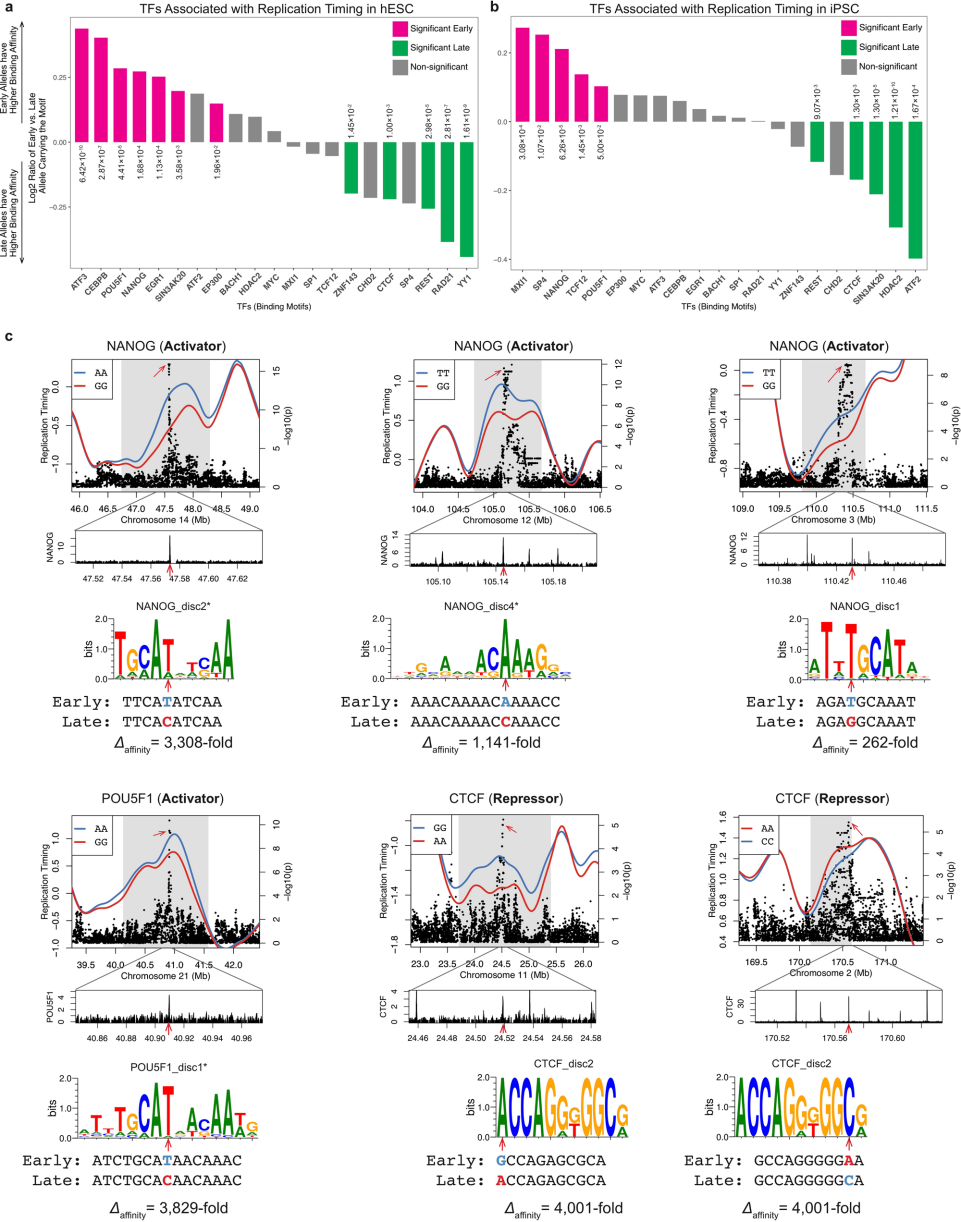
rtQTLs affect replication timing by altering TF binding motifs. **a**, **b** Binding of TFs such as OCT4 and NANOG promotes earlier replication, while binding of CTCF, REST and other factors is associated with later replication in hESCs (**a**) and iPSCs (**b**). Chi-squared test, FDR <10%. **c** Examples of rtQTLs altering binding affinity of TFs that function as replication activators or repressors. Heterozygous profiles are not plotted. Center panels: ChIP-seq tracks. Lower panels: sequence logos of the motifs containing the rtQTL SNPs, motif names, and changes in binding affinity (calculated based on motif scores). Asterisk indicates that the motif was on the negative strand and the sequence shown is the reverse complement. Red arrows: locations of the rtQTL SNPs. For activators, the rtQTL allele associated with early replication encodes an intact binding motif, while the allele associated with late replication abolishes the motif. Repressors have the opposite pattern: the early allele abolishes the motif.

Transcription factors (TFs) bind DNA in a sequence-specific manner at characteristic motifs. This offers an opportunity to test, at base-pair resolution, whether TF binding affects replication timing at rtQTLs (Methods). Strikingly, OCT4 and NANOG had significantly higher binding affinity for early- compared to late-replicating alleles in both hESCs and iPSCs, while EP300 and ATF3 (Activating Transcription Factor 3, which is enriched at EP300 sites^51^), were linked to early replication at least in hESCs (Fig. 5a, b). These associations appeared to be independent from gene expression, as they were reproduced for rtQTLs > 250 kb away from expressed genes. For these early-replication-associated TFs, the rtQTLs fell within the TF binding motifs such that a single base-pair change disrupted or even abolished binding; this was associated with delayed replication of the rtQTL-affected initiation site (Fig. 5c). An unexpected finding was rtQTL alleles with the opposite effect, i.e., higher binding affinity associated with later-replication. We infer that in these cases protein binding suppresses replication initiation (Fig. 5). These included CTCF, an insulator of topologically associated domains (TADs); REST (NRSF), a repressor of transcription^52^; ZNF143, which associates with the CTCF-cohesin cluster^53^; and at least in hESCs also RAD21 (part of the cohesin complex) and YY1, which co-localize with CTCF at TAD boundaries^54–57^. These associations were yet stronger when considering only motifs with biochemically confirmed TF binding when data were available (Methods). Since some TF motifs were inferred using ChIP-seq data, they may be motifs for co-factors, and in these cases, rtQTLs may affect the binding of the co-factor instead of the TF per se. Furthermore, TFs and the histone modifications appeared to be independent determinants of replication timing. The majority (82%) of the me^3^ac^hyper^ histone modification regions did not overlap any of the TFs that were positively associated with replication timing, and the histone modification predicted replication timing peak locations even better when it was not associated with activator TFs (median distance: 38.8 kb) than at regions overlapping TF binding (53.7 kb; random was 161.71). Taken together, we conclude that some rtQTL alleles alter DNA binding protein motifs, abolish a DNA binding site or generate a new one, and consequently alter DNA replication timing through specific TF (or co-factor) binding. This analysis uncovers several factors that can thus regulate DNA replication timing. In addition, different factors influence subsets of replication initiation sites, further illuminating the complex combinatorial landscape that controls human DNA replication timing. Finally, these results demonstrate one molecular mechanism whereby a single base-pair alteration could affect the replication timing of an extended genomic region.

## Discussion

The spatiotemporal regulation of DNA replication, and its dependence on regulatory DNA sequences, are poorly understood. Here, we leveraged population-scale replication timing and genetic polymorphism data to perform the equivalent of millions of surgical genetic interrogations of replication timing determinants. This approach enabled us to identify an unprecedented number of precise sequence determinants of replication timing.

Studying chromatin structure at rtQTL sites revealed histone modification combinations that predicts a subset of replication initiation sites across cell types. These combinations represent noncanonical functions of histone H3 lysine methylations that form a previously undescribed bivalent chromatin state^27^ present at localized sites throughout the genome. Prior biochemical evidence supports an involvement of these histone marks in DNA replication initiation^13,32–37,39–42^. We propose that these histone modifications promote local replication activity, although we do not necessarily imply that they mark the locations of replication origins per se. We suspect that there could be various such combinations of histone modifications, spanning localized chromosomal regions, that influence the propensity of replication initiation. Further studies with yet more comprehensive replication timing and chromatin data could refine and expand the associations between the epigenome and replication timing regulation.

rtQTLs further associated with inter-individual variation in histone marks and TF binding affinity. In many cases, several *cis*-acting sequences affected a region’s replication timing both proximally as well as distally. Altogether, we were able to assign at least one molecular mechanism to 98.8% of rtQTLs, while two or more determinants were implicated in 93.9% of rtQTLs (Supplementary Figure 10). Replication timing determinants acted additively among nearby sequences, synergistically between histone modifications, and modularly across transcription factors. This system generates a continuum of replication activities: some epigenetic marks may contribute only modestly to replication activity, or even suppress it, yet can interact with other factors to ultimately promote robust early replication. Taken together, this study systematically reveals a complex, combinatorial landscape of genetic regulation of human DNA replication timing.

A recent study using CRISPR/Cas9-mediated deletions in mouse ESCs identified several interacting sequence elements responsible for early replication (“early replicating control elements”^11^). Consistent with our results, the identified elements bound P300 and pluripotency-related TFs. However, the specific features identified with deletions represented the properties of only 1.5% of rtQTLs. Instead, rtQTLs associated with replication throughout S phase (not just with early replication); some interacted with others while many did not; and there was no single DNA-binding factor that was always bound to them. rtQTL mapping reveals a much more complex picture of replication timing regulation than previous approaches were powered to uncover. Replication timing regulation emerges as a quantitative trait, requiring a quantitative genetics approach to elucidate its complex sequence underpinnings. rtQTL mapping in larger sample sets and additional cell types will further refine the details of replication timing regulation and reveal additional *cis*-acting sequences and their mode of action.

Our findings draw corollaries between replication timing regulation and classical concepts of gene expression regulation: promoter/enhancer logic, activators and repressors, and combinations of histone modifications. Thus, replication and transcription regulation appear to be based at least in part on similar principles and building blocks. Replication timing is robustly encoded in DNA, yet multiple DNA sequences dictate DNA replication combinatorially via chromatin effectors. The replication timing program of the human genome emerges as being sequence-dependent, without being sequence-specific.

## Methods

### Ethics statement

All human subjects research was reviewed and approved by the Columbia University IRB and by the Columbia embryonic stem cell research committee. All human subjects provided informed consent and oocytes and sperm were donated for research purposes after informed consent. The IRB reviewed the use of gametes and embryos for the generation of ESC lines, including whole genome sequencing and the deposition of sequencing data to public databases. Gamete or embryo donors provided informed consent for genetic research and all samples were de-identified. There was approval and consent for the collection of tissue samples and derivation of iPSCs. ESC and iPSC lines derived prior to the NIH 2015 genomic data sharing policy did not include (and did not require) specific informed consent for whole genome sequencing and controlled access data sharing. MR14-20 and CUES2-6 were derived from 2015 and onwards, and included specific informed consent for whole genome sequencing and genomic data sharing.

### Whole-genome sequence data

Whole-genome sequence data and genotype calls for 121 hESC lines were obtained from Merkle et al.^19^. We denote these as “Merkle_batch1”. Nine additional hESCs were sequenced in another batch from Merkle et al.^19^, denoted as “Merkle_batch2”. We further used whole-genome sequence data from 326 iPSC lines from the HipSci Project^20^ (ENA accession number: PRJEB15299), denoted as “HipSci”. We sequenced an additional 15 hESCs and 17 iPSCs (dbGaP accession number: phs001957), denoted as “in_house_hESC” and “in_house_iPSC”, respectively.

For the in-house datasets, DNA was extracted using the MasterPure Complete DNA and RNA Purification Kit (Lucigen). Sequencing libraries were prepared using the Illumina TruSeq PCR-free kit and sequenced on an Illumina HiSeq X Ten to ~16-fold coverage with 150 × 2 paired-end reads. Sequencing was performed at GeneWiz (South Plainfield, NJ). Reads were aligned to GRCh37 using BWA, and genetic variants were called following the GATK Best Practices. Variants were filtered using GATK’s variant quality score recalibration, such that SNPs had a 99.9% sensitivity to true variants (HapMap 3.3 and Omni 2.5M)^58^ and a 99.0% sensitivity to true indels (Mills / 1000 Genomes indels)^59,60^.

### Cell culturing

#### iPS derivation

Two-milliliter Skin biopsies were made using the AccuPunch Biopsy kit (Acuderm Inc.) after local anesthesia using lidocaine. Biopsies were dissected in 4-8 pieces and placed under a glass coverslip held to the bottom of a six-well dish using silicon grease in DMEM medium containing 10% FBS (Atlanta Biologicals #S11150) and Anti-Anti (Thermo Fisher 15240112). After 1 month, fibroblasts were dissociated, plated on new 6-well dishes in the same medium, and after reaching confluence, frozen at passage 2. Reprogramming was performed using the mRNA and miRNA booster reprogramming kits of Stemgent (now Reprocell) using sequential mRNA transfections for 10 days, according to manufacturer instructions. Single colonies were picked manually and passaged for expansion, cell line characterization, and cryopreservation. The culture of human iPS cells for the preparation of genomic DNA were cultured on Geltrex (Thermo Fisher #A1413302) in StemFlex medium (Thermo Fisher # A3349401).

#### ESC derivation

Human embryonic stem cells (ESC) were derived as previously described^61,62^. Briefly, the trophectoderm of blastocysts was ablated using 20-40 laser pulses of 400ms at intensity set to 100% (Hamilton Thorne). The inner cell mass was plated on irradiated mouse embryonic fibroblast feeders (Globalstem), DMEM HG (Thermo Fisher #10569010), Glutamax (Thermo Fisher #35050079) and PenStrep (Thermo Fisher #15140163) and 18% KOSR (Thermo Fisher #10828010) and 10ng/ml bFGF (Thermo Fisher #13256029), Rock inhibitor Y-27632 (Selleckchem #S1049) and embryonic stem cell grade FBS (Hyclone). After ESC outgrowth became visible, Rock inhibitor was phased out, and passaged manually for one to two passages in the same media. Thereafter, ESCs were passaged enzymatically using TrypLE Express (Thermo Scientific #12605036) and were cultured on Geltrex (Thermo Fisher #A1413302) in StemFlex medium (Thermo Fisher # A3349401). Blastocysts were obtained after ICSI of donated oocytes and sperm.

### Inference of DNA replication timing

DNA replication timing was inferred by analyzing sequence read depth (corrected for GC content bias) in non-overlapping windows of 10 kb of uniquely alignable sequence using GenomeSTRiP^18,63^. Among the 121 hESC lines from Merkle_batch1, five did not optimally thrive in culture, resulting in read depth profiles with low correlations to other samples; these cell lines were excluded from further analysis. We excluded 26 of the 326 iPSC lines from the HipSci dataset for the same reasons. As described below, further filtering were performed for Merkle_batch1 and HipSci datasets. Replication timing inference for the in-house datasets is described separately (see the “validation of rtQTLs” section below).

We first filtered out genomic windows for which the DNA copy number is influenced by mapping inaccuracies, technical outliers or regions likely to be CNVs in our data. We removed all windows that: (1) spanned GRCh37 gaps; (2) overlapped structural variants (SV) with ≥ 1% MAF in the 1000 Genomes European individuals; (3) overlapped short CNVs (median size: 3.51 kb) identified directly in the analyzed cell lines (applicable for Merkle_batch1 only, Merkle et al., submitted^19^). Filters (2) and (3) remove polymorphic copy number regions that could both confound the inference of replication timing as well as potentially lead to the false association of SNPs with CNVs/SVs instead of true replication timing variants. (4) There were still genomic regions remaining with consistently extreme copy number values even after exclusion of mapped CNVs. These regions typically corresponded to pericentromeric regions, segmental duplications, and other genomic regions with poor reference sequence continuity. To identify these windows, we calculated the median value (across samples) of each genomic window. We then used the median of these values to represent the “common” copy number of the genome. Any window with a median of more than 0.4 copies above or below this common number was removed. (5) Similar to criterion 4 but using the 25%/75% percentile instead of median, which allowed to better capture outliers. Specific parameters for criteria 4 and 5 (as well as the filtering steps described below) were chosen based on extensive testing and are optimized to be the least disruptive to the data as possible, learning towards stringency. Altogether, 28,769 data windows (11.0% of all windows) were removed, leaving 232,027 windows after filtering for Merkle_batch1. For the HipSci dataset, 239,516 windows remained.

We further removed, in individual cell lines, genomic windows that were copy number outliers in specific samples (rather than across all samples). We removed data points that were at least 0.6 copies above or below the common copy number (see above), or at least 0.25 copies above or below the median copy number of that replication timing window, in any particular sample. Together, these two parameters ensured the efficient filtering of absolute or relative outliers, respectively. We also removed data points that were in a large CNV (median size: 3.02 Mb) identified in that individual (applicable for Merkle_batch1 only). These CNVs are predominantly of cell culture origin and not shared with other samples, thus the reason to remove them from the applicable samples rather than from the data in general. In addition, we used correlations and autocorrelations to identify samples in which an entire chromosome or chromosome arm appeared to be subclonally duplicated or deleted (sub-integer change in copy number over a large region). These criteria were implemented to further remove outlier data points. Data after the above filtering steps are referred to as “filtered raw data”. We provide a visual demonstration to clarify and justify the parameter choices (Supplementary Fig. 1a). Our filtering criteria are effective in removing CNVs and outlier data points, resulting in DNA copy number profiles optimal for replication timing inference.

Processing of the X chromosome data was performed separately for males and females. For males, because they only carry one X chromosome, all the thresholds above were divided by two.

The filtered raw data was further normalized to *Z*-score (i.e., autosomal mean of zero and standard deviation of one) by subtracting the mean then dividing by the standard deviation of all data points, and smoothed using a penalized smoothing spline using the R pspline package with smoothing parameter 10^-16^ (chosen after testing multiple parameters to be the most representative of the underlying raw data; this parameter is also consistent with previous papers)^18,64^. For each chromosome, we smoothed across gaps only if the gaps were shorter than 300 kb. Continuous genomic segments (between gaps) that were smaller than 300 kb were removed from further analysis, since smoothing of these short segments was sensitive to individual outlier data points and to edge effects. Data after the above normalization and smoothing is referred to as “smoothed data” (Fig. 1a) and was used in further analyses. The total length of replication timing windows in the smoothed data was 2330.66 Mb for autosomes (referred to as the “analyzable genome”), 121.15 Mb for the X chromosome in females, and 121.19 Mb for the X chromosome in males. For analyses involving the analyzable genome, only autosomal rtQTLs were counted.

For correlation calculations involving sib pairs vs. non-sib pairs (Merkle_batch1) and cell lines derived from the same donor vs. different donors (HipSci), we used replication timing data from all autosomes. The Wilcoxon rank-sum test was used to assess significance. For the analysis regarding IBD segments in sib pairs, we first inferred pairwise IBD using TRUFFLE^65^, then binned the IBD segments into 2.5 Mb regions. The purpose was to minimize bias in correlation estimation because of variable IBD segment sizes. We calculated pairwise correlation in these regions, and assigned the estimate to one of three groups (IBD 0/1/2). ANOVA was used to assess the significance of the difference in average correlation among IBD 0/1/2 groups. For all box plots in this study, the center line represents median, box limits represent the first and third quartile, and the whiskers represent the maximum and minimum. Outliers as determined by the R boxplot function were not plotted. The correlation was calculated using the corr function in the Statistics::Basic Perl module. Pearson correlation of replication timing among samples and with previous Repli-Seq measurements were calculated in R. The R lme4 package was used for variance component analyses. Linear mixed-effects model was fitted for each replication timing window, with the following variables: donor, sex, age, and technical factors (Pluritest pluripotency score and novelty scores, culturing media, date of derivation, and method of derivation). When assessing the effect of rtQTLs, we also included rtQTL genotype in the model, and partitioned the donor effect into two parts: those explained and those not explained by rtQTLs.

Of note, all statistical tests used in this study were two-sided unless otherwise noted. Details of statistical methods used in this paper are listed in Supplementary Table 2.

### Identification of replication timing peaks

We identified peaks in the Merkle_batch1 dataset. For each sample, peaks were identified in the replication timing profile as local maxima. Peaks across all samples were then clustered using agglomerative hierarchical clustering in MATLAB (functions linkage and cluster) with a distance threshold of 200 kb, which yields a list of peak clusters, each containing one or more peak locations. When a cluster contained multiple peaks from the same sample, the peak closest to the cluster center was retained and all other peaks from the sample were dropped. For each peak cluster, the boundary was defined as the full range of peak locations in this cluster for hESCs, and the 5th to 95th percentile of peak locations in the cluster for iPSCs (to account for the more common presence of outliers in the iPSC dataset). We only used peak clusters that contained peaks from more than 10% of the samples.

### Identification of replication timing variants

We searched for replication timing variants using the Merkle_batch1 and HipSci datasets. We expect genomic regions with significant replication timing variation among samples to have greater standard deviation (SD) in their replication timing across samples. We calculated the SD of replication timing across the genome, excluding windows spanning genome gaps, and identified local peaks in the smoothed SD data. Peaks with SD values greater than the genome-wide SD mean were called as replication timing variants. To evaluate the significance of these tentative variant regions, we performed pairwise *t*-tests on raw replication timing data for all pairs of samples in 500 kb windows centered at the called variant peaks. We required variants to be significance at the 0.05 levels after Bonferroni correction for the number of sample pairs and the number of variant regions tested.

In a second step, we extended each replication timing variant until cross-individual replication timing variation fell below significance. Specifically, we tested adjacent 200 kb windows, sliding 100 kb at a time, extending until there were less than 0.1% significantly different cell line pairs. In cases where replication timing variants overlapped each other after extension, they were merged if the correlation of replication timing across samples at the SD peaks was greater than 0.9, and otherwise separated at the SD valleys (local minima) between peaks. Finally, we removed replication timing variants on chromosome 19 due to a high false-positive rate related to the extreme GC content and therefore noise in the data on this chromosome. We further removed variants driven by no more than 1% of the samples.

### Data processing prior to rtQTL mapping

#### Sample selection

We performed principal component analysis (PCA) on the genotypes of the hESC lines, using the 1000 Genomes Phase 3 European, East Asian, and African samples as references. Eight samples appearing to have non-European ancestry (admixed or East Asian) were removed from rtQTL mapping, leaving 108 individuals for further analysis. PCA was performed using the SNPRelate package in R^66^. We also performed PCA with the HipSci dataset, and confirmed that all samples were of European ancestry. A total of 192 unrelated samples in the HipSci dataset were used for rtQTL mapping. While we kept sib pairs in the ESC dataset, all rtQTLs in ESC were reproducible (at nominal *p* < 0.05) when using only unrelated samples.

#### Genotype imputation

Imputation was performed with IMPUTE2^67^ using the 1000 Genomes Project Phase 3 reference panel and default parameters. Variants with minor allele frequency (MAF) ≤ 1% in Europeans or Americans were not used for imputation. Imputed variants with average genotype probability ≥ 80% were used in subsequent analyses.

Prior to rtQTL mapping, we filtered out variants that had MAF < 5%, were nonbiallelic, or that deviated from Hardy-Weinberg equilibrium (*p* < 1×10^-3^). In addition, we required that variants should have all three genotypes (homozygous reference allele, homozygous alternative allele, and heterozygous genotypes) observed in the samples.

#### PCA of replication timing data

To account for potential batch effects and other unknown systematic biases in the replication timing data, we performed PCA using the filtered raw data with R function prcomp. Principal components (PCs) of the filtered raw data (“phenotype PCs”), along with the genotype PCs calculated above, were used as covariates in rtQTL mapping.

### rtQTL mapping

#### Selection of phenotype PCs in rtQTL mapping

We followed the eQTL mapping framework used in the GTEx Project^24^ (https://gtexportal.org/home/documentationPage) to map rtQTLs. We included the genotype (first three, similar to GTEx) and phenotype (first *k*) PCs in rtQTL mapping to account for genetic and non-genetic confounding factors, respectively. To find the optimal *k*, we tested each integer from 1 to 40. We consider the optimal *k* as the one leading to the highest number of windows harboring rtQTLs identified in rtQTL mapping. In this analysis, permutation parameter “permut 50 500” was used in fastQTL. Window level *p* values were calculated, and the R package *q* value^68^ was used to identify windows harboring rtQTLs at 10% FDR. This resulted in 24 and 22 selected as the optimal *k* for ESC and iPSC rtQTL mapping analysis, respectively, which was used in all subsequent rtQTL mapping analyses.

#### Cis-rtQTL mapping using fastQTL

We implemented a two-step approach to map rtQTLs using fastQTL^69^. We generally restricted our analysis to *cis*-rtQTLs, defined as 1 Mb upstream or downstream of the center of each tested replication timing window. The 1 Mb distance threshold is standard in eQTL mapping studies and allows capturing more distal *cis* effects (Fig. 1m). The first three genotype PCs and first 24 or 22 (for ESC and iPSC, respectively) phenotype PCs were included as covariates.

In the first step, we calculated a window-level *p*-value for each replication timing window using fastQTL, and then identified “significant windows”, i.e., windows with at least one significant rtQTL at 10% FDR, using the R package *q* value. This step is analogous to the identification of “eGenes” in eQTL mapping. For each window, fastQTL computes the lowest variant-level *p*-value and uses permutations to calculate the probability of observing a variant with equal or lower *p*-value under the scenario of no association, followed by beta approximation. Adaptive permutation parameter “permut 1000 10000” was used (similar to GTEx). We also repeated this step at 5% FDR.

In the second step, we identified genetic variants (referred to as SNPs for simplicity) associated with the “significant windows” identified in step 1, at 10% FDR. Here, we used a permutation-based strategy to determine the significance threshold for each tested window. By definition, FDR is the ratio of false positives (FP) to the sum of FP and true positives (TP). At a given *p* value threshold *p*_*t*_, variants passing *p*_*t*_ are composed of both TP and FP. However, if we permute the phenotype, all variants with *p*-values lower than *p*_*t*_ are FP. Therefore, for a given window, FDR for a given *p*_*t*_ could be estimated as the mean number of variants passing *p*_*t*_ in permutations (i.e., all FP) divided by the number of variants passing *p*_*t*_ in the true association test (FP+TP). We then consider the maximum *p*_*t*_ with FDR ≤ 10% as the significance threshold of the window. The mean number of variants passing *p*_*t*_ in permutations was computed based on 500 permutations.

#### Evaluation of inflation of rtQTL mapping

To ensure that the computed variant-level *p*values were not inflated, we calculated inflation factor with the Genomic Control method^70^. We selected 200 windows (100 selected from windows carrying putative rtQTLs, and the other 100 randomly selected from the rest of the genome) and computed their association with genome-wide variants. We obtained variant-level statistics (which follows ${\chi }_{1}^{2}$ distribution under the null hypothesis) and computed the ratio of their median to the median of ${\chi }_{1}^{2}$ (0.456) as the genomic inflation factor. We calculated a genomic inflation factor (*λ*) as 1.03 and 1.00 for the ESC and iPSC dataset, respectively, thus the nominal *p*-values were not inflated; this was also supported by quantile-quantile plots.

#### Identification of rtQTLs

The following procedure was used to identify discrete rtQTLs, i.e., independent (not in LD) association signals, based on the significant SNPs mapped using the aforementioned two-step approach. For clarity, we denote independent association signals as rtQTLs, each of which contains multiple SNPs that are part of the association signal.

For each window, we identified all SNPs (if any) that passed the significance threshold. We selected the SNP with the lowest *p*-value as the “tag” variant of an rtQTL and assigned SNPs in LD (*r*^*2*^ ≥ 0.2) with the tag variant to the rtQTL. If there were any SNPs remaining that passed the significance threshold, we selected the SNP with the lowest *p*-value among the remaining SNPs as the tag variant of a new rtQTL and assigned all variants in LD with the new tag variant to the new rtQTL. This step was repeated until no variants passing the significance threshold were left. For the rtQTLs identified above, we kept only those with at least 10 variants and for which the *p*-value of the tag variant was less than 10^-3^.

For all calculations involving LD, data from the 1000 Genomes Phase 3 Europeans was used whenever available. For SNPs not called in the 1000 Genomes dataset, the current dataset was used for LD calculation.

Since nearby replication timing windows are highly correlated, the same rtQTL can be detected across multiple windows. We consolidated association signals detected in different windows if they satisfy all of the following three criteria: (1) the tag variants are in LD (*r*^*2*^ ≥ 0.2), (2) the replication timing windows are correlated (*R*^*2*^ ≥ 0.1), and (3) the distance between the windows is less than 2Mb.

In addition to separating rtQTLs by LD, we subsequently performed conditional association for each identified rtQTL. For a given rtQTL, we tested the association between each genetic variant in this rtQTL and replication timing using fastQTL. We included as covariates the top genetic variant of this rtQTL as well as genotype and replication timing PCs. If a genetic variant had *p* < 0.05 after Bonferroni correction (based on the number of rtQTL genetic variants in the given rtQTL), this rtQTL was divided into multiple rtQTLs, each representing an independent association signal. rtQTLs identified using both approaches (LD and conditional analysis) were reported.

We attempted wide ranges of values for the thresholds used in this section, and found that the rtQTL results were highly robust to the choice of thresholds. We used three parameters to define independent regions and genetic variants. We considered two SNPs in LD if their *r*^*2*^ was greater than *a* (set to 0.2). We consolidated rtQTL-associated replication timing windows if their correlation *R*^*2*^ was greater than *b* (set to 0.1) and they were no more than *c* (set to 2 Mb) apart. We first fixed parameter *c* = 2 Mb and tested various combinations of *a* and *b* (0.1, 0.2, 0.5, and 0.8). As shown in Supplementary Table 3, the proportion of the genome associated with ESC-rtQTLs, the average size of rtQTL associated regions, and the mean number of rtQTLs at multi-rtQTL regions were highly robust to the choices of *a* and *b*.

We then fixed *a* = 0.2 and *b* = 0.1, and tested various choices of *c* (0.5, 1, 2, 3, 4, 5 Mb). We did not test *c* < 0.5 Mb because replication timing windows show high correlation (*r* > 0.6) when they are less than 0.5 Mb apart. As shown in Supplementary Table 4, the rtQTL results were also highly robust to the choice of *c*.

In rtQTL mapping, we also discarded putative rtQTLs that were supported by less than 10 genetic variants or if the minimum *p*-value was greater than 10^-3^. These two criteria were designed to filter out very weak statistical associations (median number of variants supporting an rtQTL was 133, and median *p*-value for both ESC and iPSC rtQTLs was on the order of 10^-6^). Therefore, the use of these two filters does not result in overestimation of genomic regions associated with rtQTLs or the number of independent rtQTLs per region.

#### Filtering of rtQTLs

The putative rtQTLs identified were subjected to further filtering. First, we determined the boundaries of regions that significantly associated with each putative rtQTL. Starting at the window that most strongly associated with the tag variant (i.e., with the lowest *p*-value) of an rtQTL, we extended the region bi-directionally until the association was no longer significant (*p* > 0.05). We refer to this region as the “associated region”.

Next, we filtered false positives suspected to be potentially caused by short CNVs. During data processing (described above), we removed windows in which copy number measurement are potentially influenced by CNVs. However, short CNVs, spanning only one or two windows, may not have been detected and filtered and could lead to false-positive rtQTLs (if they are in LD with SNPs). This type of false positive was identified by utilizing the raw unsmoothed data as follows: if a putative rtQTL is a false positive caused by a CNV, the association signal would be (1) only observed in a small number of unsmoothed raw windows (overlapping with the CNV), and (2) will be more strongly associated with the raw data than with the smoothed data (in which the CNV will be smoothed within a broader region, thus decreasing association).

We computed the association *p*-values of the tag variant of each rtQTL with the (1) smoothed data within the associated region, (2) filtered raw data within the associated region, and (3) data that were removed during data processing within 1 Mb upstream or downstream of the associated region (referred to as “removed data” below).

Putative rtQTLs must satisfy all of the following criteria to be included in the final list of rtQTLs:

1. In the raw data, the tag variant must be associated (*p* < 0.05) with at least five windows. This removes short CNVs that were not filtered during replication timing processing. The majority of such CNVs are no longer than five windows (50 kb or less).
2. The minimum *p*-value of the raw data must be higher (i.e., less significant), or no more than one order of magnitude lower, than that of the smoothed data. This further removes CNVs that are minimized by smoothing but can still be present and lead to false associations (which would be stronger for unsmoothed data). We found that a cutoff of one order of magnitude of significance performs well without over-removing non-CNV data.
3. The minimum *p*-value of the removed data must be higher, or no more than one order of magnitude lower, than that of the raw data. This criterion is relaxed to two or four orders of magnitude for rtQTLs with top *p* value ≤ 5×10^-6^ and ≤ 5×10^-8^, respectively. We further found that some windows removed as CNVs during the replication timing data processing did not encompass the full CNV length and still led to false rtQTL associations. To mitigate those, we further required that the minimum *p*-value of such removed data must be higher, or no more than one order of magnitude lower, than that of the raw data. This is similar to criterion (2) above. Since the fluctuations in -log_10_(*p* value) scaled with rtQTL association strength, we relaxed this criterion to two or four orders of magnitude for rtQTLs with top p-value ≤ 5×10^-6^ and ≤ 5×10^-8^, respectively. These numbers were optimized empirically. Similarly, to further eliminate rtQTLs caused by residual unremoved windows caused by CNVs, we required that no more than two windows in the removed data have *p*-values lower than the minimum *p* value for the raw data. This criterion is relaxed to three windows for rtQTLs with top *p* value ≤ 5×10^-8^.
4. Finally, we sought to remove very weak rtQTL associations that are less likely to be true. We therefore required that the minimum *p*-value from the raw data must be less than 0.01 and that the associated region must be larger than one replication timing window.

In total, we identified 608 ESC rtQTLs, among which 603 were on autosomes and five were on the X chromosome in males. No rtQTLs were found on the X chromosome in females. This was not due to the reduced number of individuals tested, but likely resulted from the less structured replication timing profiles attributed to the female inactive X chromosomes: the similar-sized chromosome 7 had ten rtQTLs in the 50 male samples, not significantly different than the male X chromosome (*p* = 0.31, Fisher’s exact test), while there were fifteen rtQTLs on chromosome 7 in 66 female samples, significantly more than the none found on the female X chromosome (*p* = 7.41×10^-5^). We identified 1167 iPSC rtQTLs. The nominal *p*-value of rtQTLs ranged from 1.02×10^-69^ to 9.63×10^-4^ (106 and 218 rtQTLs [17.4% and 18.7%] had *p* ≤ 5×10^-8^ in the ESC and iPSC dataset, respectively). The early- and late-replicating alleles were equally likely to be the reference allele (binomial *p* = 0.55), thus rtQTL mapping was not influenced by reference mappability bias.

We also re-performed downstream analyses using the list of putative ESC rtQTLs without any filtering and were able to reproduce (*p* < 0.05 after multiple testing correction) the key results in the original manuscript, including all histone mark enrichments at rtQTLs and the me^3^ac^hyper^ histone modifications (as in Supplementary Fig. 4 and Fig. 3). The histone modifications were also reproduced in iPSCs and LCLs. Twelve of the 15 histone marks that show association with early or late replication (Fig. 4) were reproduced. In addition, 10 of the 12 TFs that positively or negatively associate with early replication (Fig. 5) were reproduced. Thus, our results are not sensitive to the choice of parameters in rtQTL filtering. We nonetheless noticed that the findings were generally quantitatively weaker (e.g., 22 of the 24 enriched histone marks had lower fold-enrichments) when using all putative rtQTLs without filtering, suggesting that the filtering was necessary to reduce artifactual rtQTLs in the dataset.

#### Prioritizing causal genetic variants

For each rtQTL, CAVIAR^22^ was used to produce a shortlist of possible causal SNPs at 90% probability, from all SNPs in LD with the tag variant of the rtQTL (*r*^*2*^ ≥ 0.2). The shortlisted SNPs were used in all enrichment analyses.

#### Merging ESC and iPSC rtQTLs

We integrated ESC and iPSC rtQTLs for a number of analyses. To minimize double counting of rtQTLs discovered in both datasets, we generated a merged rtQTL list for these analyses. This list excluded iPSC rtQTLs that met the following criteria: (1) a genetic variant that belongs to the given iPSC rtQTL and has a *p*-value no more than two orders of magnitude higher than the top *p* value of the iPSC rtQTL also belongs to a ESC rtQTL, and (2) the direction of effect of the given genetic variant is the same in the iPSC and ESC datasets. We merged the 608 ESC rtQTLs and 1167 iPSC rtQTLs into a list of 1,617 combined rtQTLs.

We found that the above integration scheme was highly robust. We tested a new integration scheme, by excluding iPSC rtQTLs if any genetic variant in LD (*r*^*2*^ > 0.2) with their top genetic variant was in LD with the top genetic variant of any ESC rtQTL. This is likely the most conservative approach for integrating ESC and iPSC rtQTLs. Using this scheme, we identified 1564 rtQTLs associated with 31.4% (731.1 Mb) of the genome. These estimates were very similar to the estimates using the original approach (1617 rtQTLs, 31.8%/741.8 Mb), which suggests that the approach described above was appropriate and did not overestimate the effects of rtQTLs.

#### Factors driving the increased rtQTL calling ability

We identified 1,617 rtQTLs in hESCs and iPSCs, which was two orders of magnitudes more than our previous study.^18^ Several factors conspire to provide the much larger number of rtQTLs identified in this study.

First and foremost, the quality of the data is much greater in the current study. This is mostly driven by the deeper sequence data (~30x sequence coverage, compared to 2–4x coverage in our 2014 study). In addition, ESCs and iPSCs are more proliferative than LCLs, leading to stronger replication signal in the sequence data.

To demonstrate the effect of data quality on rtQTL mapping, we artificially added noise to the iPSC replication timing data from this study to match the level of noise in our 2014 study. We did this for chromosomes 2, 3, and 4 as a representative data subset and evaluated noise using autocorrelation of raw replication timing data along chromosomes. After adding noise (Supplementary Figure 1c), the number of rtQTLs identified on the three chromosomes dropped substantially, from 282 to 17 (~6%).

A second critical factor enabling the enhanced rtQTL identification in the current study is our improved analytical framework. In our previous study, we were very strict in identifying only the strongest replication timing variants, and we only performed rtQTL testing on those. In contrast, we aimed here to identify as many rtQTLs as possible under the statistical power constraints. Limiting our current search to the same criteria we used in the previous study led to a 3.8-fold decrease in the number of rtQTLs identified (from 603 to 158 for hESC rtQTLs). Other factors contributing to the increased number of identified rtQTLs are an improved two-step rtQTL mapping approach, and more optimal calibrations of principal component (PC) regression for replication timing and genotype.

### Validation of rtQTLs

To validate the iPSC rtQTLs, we examined their reproducibility in the Merkle_batch1 ESC dataset (108 European ancestry samples only). Validation was performed using fastQTL^69^ by testing the association between the strongest rtQTL SNP and the replication timing locus closest to the locus with the strongest association in the discovery set (HipSci iPSCs). Three genotype PCs and 24 phenotype PCs were included as covariates. When the strongest rtQTL SNP was not available in the validation dataset (Merkle_batch1 ESCs), an rtQTL SNP from the same rtQTL that has *p* value less than two orders of magnitude higher than that of the strongest rtQTL SNP was used instead. We found that the -log_10_(*p*-values) of rtQTLs are highly correlated between the discovery and validation datasets (Pearson *r* = 0.75, *p* = 1.28×10^-176^). We then repeated this analysis in the opposite direction (validate ESC rtQTLs using HipSci iPSCs) and obtained similar results (Pearson *r* = 0.76, *p* = 7.81×10^-113^). These observations support that the rtQTLs identified in this study are highly reproducible.

We also used three additional datasets to validate ESC rtQTLs. The first dataset contains 9 hESCs in Merkle_batch2 and the 8 hESCs in Merkle_batch1 that were excluded in rtQTL mapping due to ancestry. The second and third datasets are the in-house hESC and iPSC dataset, respectively.

For the first dataset, validation was performed in fastQTL. Validation using the second and third datasets were performed in MATLAB by calculating the Pearson correlation *p*-value between the strongest rtQTL genetic variant and the replication timing locus with the strongest association in the discovery set. We tested rtQTLs of which the top genetic variant was polymorphic and had all three genotypes in the validation dataset. rtQTLs were excluded if the alternative allele of the top genetic variant in the validation dataset was not consistent with that of in the discovery set. This left 427 regions that could be tested in the third dataset, and 396 regions in the fourth dataset. Replication timing of these two datasets were inferred using GenomeSTRiP (as described above) in 2.5 kb windows of uniquely alignable sequence^63^. For each sample, windows with copy number > 3 or < 1 were removed. We used a segmentation algorithm (segment in MATLAB) to further remove outlier data points (segments with mean > 2.45 or < 1.55 were removed). The data were then smoothed using a cubic smoothing spline with parameter 10^-17^.

We considered an rtQTL as “validated” if it was associated with replication timing with nominal *p* < 0.05 and had the same direction of effect in at least one of the validation datasets. The binomial test was used to assess significance of the number of validated rtQTLs, with binomial parameter calculated as 1–(1–0.05/2)^4^ = 0.0963 (i.e., the probability under random chance that an rtQTL will be validated in at least one dataset).

#### SMARD

SMARD analysis was carried out as previously described^23^. Briefly, cells were pulse-labeled sequentially with 25 μM IdU and CldU. The cells were then embedded in 1% InCert agarose and lysed. The remaining embedded genomic DNA was digested with restriction endonucleases. Pulsed-field gel electrophoresis (PFGE) was used to separate DNA according to size. The segment containing the locus-of-interest was identified with Southern blot and the gel slice was excised. Agarose was then melted, and individual DNA strands were stretched on silanized glass slides. Immunostaining was employed to detect the halogenated nucleotides in the replicated DNA. Biotinylated FISH probes were used to identify DNA molecules containing the locus-of-interest.

### Multi-rtQTLs

To identify multi-rtQTL regions, we considered separate rtQTLs to be associated with the same region if the replication timing loci most strongly associated with them were correlated (*R*^*2*^ ≥ 0.2) across individuals, were in physical proximity (<2 Mb apart), and each provided additional explanatory power for replication timing. Secondary rtQTLs were either not in LD with the primary ones (130 and 265 multi-rtQTL regions in the ESC and iPSC dataset, respectively), or provided additional explanatory power despite being in LD (5 cases in ESC and 10 cases in iPSC).

We found that the distance threshold (2 Mb) used in multi-rtQTL identification was robust. As described above, we discovered 135 multi-rtQTL regions in the hESC dataset. We tested various values for *d* (0.25, 0.5, 1, 2, 3, 5, 10 Mb), and found that the number of multi-rtQTL regions identified was stable to change in *d* (Supplementary Table 5). Specifically, when we set *d* = 10 Mb, though we identified multi-rtQTL regions with up to eight rtQTLs, there was only a modest increase in the average number of rtQTLs per multi-rtQTL region (12%) and total number of multi-rtQTL regions (11%).

Some analyses were performed with ESC and iPSC multi-rtQTL regions combined. To avoid double-counting in these analyses, we excluded iPSC multi-rtQTL regions that has at least one rtQTL that was also found in the ESC dataset. We combined 135 ESC and 275 iPSC multi-rtQTL regions into 318 multi-rtQTL regions.

We examined the possible interaction between primary and secondary rtQTLs in regions with two, three, and four rtQTLs. If there was no interaction, we expect that the replication timing in these regions will be positively linearly correlated with the dosage of early-replicating alleles. To enable pooling of multi-rtQTL regions for Fig. 2e, f, we normalized replication timing for the loci with the strongest association with the primary rtQTL of each multi-rtQTL region to *Z*-score (by subtracting the mean and dividing by the standard deviation of replication timing of this locus among samples) and denoted them as relative replication timing. They were pooled and linear regression analysis was performed using the R 1m function.

We used a likelihood-ratio test to assess whether the additive or synergistic models better-explained replication timing at multi-rtQTL regions. We tested the null hypothesis by which replication timing is proportional to the number of early-replicating rtQTL alleles carried by an individual at a multi-rtQTL region (additive effect), against the alternative, by which replication timing is more extremely biased in individuals carrying multiple early (or late) rtQTL alleles (synergistic interaction). We used 58 regions that harbored two rtQTLs and had at least one individual with zero and one with four early-replicating alleles. We fitted two linear models, with the response variable being replication timing and the explanatory variable being genotype dosage. In the null (additive) model, genotype dosage was between zero to four, matching the number of early-replicating alleles that the individual carried. In the alternative (synergistic) model, genotype dosages of individuals carrying zero or four early-replicating alleles were estimated from actual data. We then compared −2×(log likelihood ratio) with the chi-squared distribution with two degrees of freedom to obtain a *p*-value.

We also tested for interactions between pairs of genetic variants in affecting replication timing using a linear model, explicitly modeling main and interaction terms. Genotypes were coded as -1, 0, and 1 for this model. *P-*values for the interaction term were extracted, followed by Bonferroni correction. We first applied this test to the discovered rtQTLs. We then applied this test to replication timing windows and genetic variants (in *cis* within 1 Mb of a given replication timing window) genome-wide. To reduce the number of tests, we pruned replication timing windows based on a correlation threshold of *r* > 0.5 and pruned genetic variants based on LD (threshold: *R*^*2*^ > 0.5), resulting in 8499 replication timing windows and 551,645 genetic variants. We note, however, that this analysis does not rule out the presence of non-additive modes of interaction.

We examined whether the primary and secondary rtQTLs in ESC were in close spatial proximity in nuclear space. We obtained Hi-C contact matrix of the H1 cell line from Juicebox^71^ and computed the contact score between each pair of primary and secondary rtQTLs. We compared the median of these scores with 100 permutations, in which the distances between primary and secondary rtQTLs were preserved but actual genomic locations were randomly shifted between 1 and 2 Mb up- or downstream. *P* value was computed using *Z* score, with mean and standard deviation estimated from the permutations. We confirmed the normality assumption using the Shapiro-Wilk test.

### Epigenetic enrichment analyses

#### Data sources

Chromatin state and histone mark data for eight human ESC lines (seven of which are included in our primary replication timing data) and five human iPSC lines were obtained from the Roadmap Epigenomics Project^72^. For overlaps with rtQTLs, we used gappedPeak data for histone modifications, validating our results with narrowPeak data (which are less inclusive, more stringently called ChIP-seq data peak locations). We used the looser definition of gappedPeak since we did not assume that all modifications necessarily appear in the exact same site; some of the histone marks implicated here (e.g. H3K36me3, H3K9me3) are diffuse, and we did not know *a* priori that there will be overlapping histone modifications associated with rtQTLs. Mammalian DNA replication origins are thought to initiate over chromosomal “regions” between several kbs to several tens of kbs, thus it is more likely that histone modifications would regulate replication initiation in a regional manner rather than at very precise locations.

For the analyses of overall enrichment of epigenetic features at rtQTL locations, we combined (i.e., took the union of) histone peaks and chromatin state calls from the eight hESC lines (for the hESC analyses) or the five iPSC lines (for the iPSC analyses). For histone marks, observed data were used when available, and imputed data (from ChromImpute^73^) was used when observed data was not available. Imputed data were used for the plotting of histone tracks. Binding site information for 51 TFs was obtained from the ENCODE Project^46^. SOX2 binding site information was obtained^49^. TFs with binding sites that overlapped <15 rtQTLs were excluded from this analysis.

#### Enrichment calculations

For each feature (chromatin state, histone marks, TF, etc.), we are interested in the number of rtQTLs that have at least one SNP overlapping with the feature, and whether this is more or less likely (i.e., enriched or depleted) than expected by chance. Statistical significance was assessed with the one-tailed binomial test. The binomial parameter *p* was estimated from 100 random permutations, from which we estimated the probability of random SNPs (matched with the rtQTLs, see details below) overlapping with the feature. Correction for multiple testing was applied when multiple features from the same category (e.g., histone marks) were tested.

For each rtQTL, we searched for random SNPs that match the characteristics of the tag variant of the rtQTL (denoted as “actual tag variant”) and used the matched variants (“matched tag variants”) to tag the random sets of SNPs used in permutations. We required that the matched tag variants must be at least 2 Mb away from the actual tag variant. The matched tag variants must also have satisfied all three following criteria: (1) have similar minor allele frequency (<5% difference), (2) have similar distances to the nearest replication initiation site and terminus (<50 kb difference), and (3) have similar replication timing (<0.5 units difference) with the actual tag variant. We require the matched tag variants to have the same number, or more, SNPs in LD (*r*^*2*^ ≥ 0.2) than the actual tag variant.

In each permutation, and for each rtQTL, we constructed a set of random SNPs using SNPs in LD with a randomly selected matched tag variant. The number of variants in the set is the same number of variants included in the actual rtQTL. Eleven (1.82%) rtQTLs in hESC and 41 (3.51%) rtQTLs in iPSC that had less than 200 matched tag variants genome-wide were excluded from the analysis.

#### Reproducing histone enrichments in individual cell lines

We initially considered the union of gappedPeak data from multiple cell lines of the same cell type, and subsequently repeated our analyses in individual cell lines and using narrowPeak data. The use of the union of data from several cell lines was due to the expectation that replication origins are affected by regional chromatin structure; that histone modification ChIP-seq data is relatively noisy; since we looked at combinations of several histone modifications appearing in the same or nearby locations; and since these analyses were centered on rtQTLs, thus the histone modifications could also be variable among the cell lines. We first re-performed the enrichment analyses of individual histone modifications at rtQTLs (Supplementary Fig. 6a and b), using the same approach as described above. We then repeated the search for histone mark combinations, in each cell line individually, using the approach as described in the section below. Following the reasonings above, and to compensate for the more stringent analysis when using individual cell lines, we did this by extending the gappedPeaks by 1 kb on both ends. We found 176 enriched three-mark combinations across six individual cell lines, 111 4-mark combinations across two cell lines, and four enriched five-mark combinations in the HUES6 cell line. The latter included the combination of H3K9me3, H3K36me3, H3K4me3, and two histone acetylation marks, as well as other combinations that all included H3K9me3, H3K36me3, and at least one acetylation mark. The four- and three-mark combinations were also enriched for H3K9me3, H3K36me3, and histone acetylations. Thus, the same histone modifications could be retrieved by analyzing individual cell lines, although, as expected, it is less specifically enriched than our original approach.

We further identified me^3^ac^hyper^ regions using histone modification data (either narrowPeak or gappedPeak) in each individual hESC line, and then calculated the distance of these regions to the nearest replication timing peak (Supplementary Fig. 6c and d), which were significantly closer to peaks than random expectation. Examples of me^3^ac^hyper^ regions at which all constituent histone marks are observed in the same hESC line are shown in Supplementary Fig. 7^1^.

### Using epigenetic features to predict replication initiation site locations

#### Identification of epigenetic feature combinations

To identify combinations of chromatin marks enriched at rtQTLs, we used a stepwise, iterative approach. The hESC rtQTLs and epigenetic data were used. We considered 29 histone marks (Supplementary Fig. 4c) and also included H2A.Z, DNase I hypersensitivity, and binding sites of 51 TFs and other DNA binding factors (referred to as TFs for simplicity).

First, we tested each individual epigenetic feature (histone mark or TF) to identify features that are enriched at rtQTL SNPs. Enrichment was examined using the same permutation-based approach described above. The only difference was that each rtQTL individual SNP was considered independently (as opposed to being considered together with other SNPs assigned to the same rtQTL), as our goal was to identify co-occurrence of epigenetic features at the same exact genomic locations. Statistical significance was assessed using Fisher’s exact test. We corrected for multiple testing at 5% FDR using the Benjamini-Hochberg correction.

Next, for each enriched feature identified in the first step, we examined whether the pairwise combination of this feature and any of the other epigenetic features have stronger enrichment. Specifically, we restricted the enrichment analysis to the rtQTL SNPs that carry the enriched feature and tested whether the additional epigenetic feature is enriched in the set of restricted rtQTL SNPs. This step was repeated iteratively, each round restricting the analysis to the enriched combinations of epigenetic features identified in the previous round, until no further enrichment was found. In Fig. 3a, combinations containing TFs were not included for simplicity and since they were not carried through to the four- and five-mark combinations.

To identify “me^3^ac^hyper^” regions, we first identified regions that carry one of the 13 five-mark combinations and kept regions that overlap with peaks from at least 11 variable acetylation marks. We merged me^3^ac^hyper^ regions that co-occurred within 10 kb. In Supplementary Fig. 5e–g, the position of initiation sites found in >10% of the samples were determined based on local maxima in the averaged replication timing profile. When calculating distances (fractional and physical), the distance was set to zero for me^3^ac^hyper^ regions that overlap with an initiation site (i.e., the interval between boundaries of the initiation sites). If a me^3^ac^hyper^ region does not overlap with any initiation site, its physical distance was calculated as the distance to the nearest initiation site boundary.

#### Receiver operating characteristic (ROC) curves

To obtain ROC curves, we randomly selected 1000 replication timing windows at replication timing peaks as positives, and another 1000 windows far (>750 kb) from the nearest peak as negatives. The distance threshold was in place to ensure that the selected negative windows would not be too proximal to peaks. We then performed prediction on these 2000 windows. If a window was within cutoff distance of a me^3^ac^hyper^ region (in LCL, we only used the three trimethylation marks [H3K4me3-H3K9me3-H3K36me3]), we predict it as “peak”. Otherwise, we predict it as “not peak”. Then we compared the predictions with the truth and counted true positive (TP), true negative (TN), false positive (FP), and false-negative (FN) windows. We calculated the true positive rate as TP/(TP+FN) and the false positive rate as FP/(TN+FP) and constructed the ROC curves. For permutations, we randomly shifted the locations of the me^3^ac^hyper^ regions between 1 Mb and 2Mb and obtained ROC curves and AUC_ROC_ based on these random intervals.

#### Replication initiation site prediction in LCLs and iPSCs

Following best practices, the me^3^ac^hyper^ histone modifications, which were discovered using hESC rtQTLs, was validated in independent datasets. Because iPSC and LCL data were not used to identify the me^3^ac^hyper^ histone modifications, we consider them as equivalent to out-of-sample replications. In hESC as well, the histone modifications were discovered based on rtQTLs but then shown to be predictive of peak locations in general; peaks with rtQTLs were excluded in the analysis of prediction performance (e.g., Fig. 3e and f).

We assessed the generalizability of the replication initiation histone modifications in LCLs and iPSCs. LCL is a cell type that has a distinct epigenetic and replication landscape from hESC lines^74–76^, and iPSCs have similar but not identical to replication timing profiles to hESCs (*r =* 0.90). Replication timing profile for the GM12878 LCL and 192 unrelated iPSCs were inferred from whole-genome sequencing data^20,48^. In ESC and iPSC, when calculating the physical distance of predicted initiation sites to actual initiation sites, we defined peak boundaries using a clustering-based approach (see above). In LCL, because of the limited sample size, we defined initiation site boundaries as 100 kb upstream and downstream of the local maxima in the replication timing profiles. For all distance calculations (ESC, iPSC, and LCL), we excluded replication timing peaks that overlap rtQTLs in any cell type, such that these calculations were not biased by the locations used for the discovery of the histone modifications. Data for H3K4me3, H3K9me3, and H3K36me3 for the GM12878 LCL was from the ENCODE Project^46^. Additional data of H3K4me3 and H3K36me3 for 18 LCLs were obtained from^47^, and merged with the ENCODE data. Imputed acetylation data for the GM12878 was from the Roadmap Epigenomics Project^72^. Histone mark data for five iPSCs was from the Roadmap Epigenomics Project^72^. If a histone modification combination location was found in one cell type (either hESC or LCL), but no combination location was found within 100 kb in the other cell type, we denoted this region as cell-type-specific. Otherwise, this region was denoted as “shared” between the two cell types.

### Identification of features associated with replication timing

#### Chromatin states and histone marks

Replication timing data was available for seven of the eight hESC lines that were analyzed in the Roadmap Epigenomics Project. Using rtQTL and epigenetic data from these seven cell lines, we designed an analysis to identify chromatin states and histone marks associated with replication timing. The rationale is that epigenetic features promoting earlier replication would be more likely to be carried by early-replicating-associated rtQTL genotypes, and vice versa for late replication. We were only able to perform this analysis for hESC rtQTLs because we did not have replication timing, genotype, and epigenetic data for the same iPSC lines.

We aggregated information from all rtQTL SNPs, except those that are monomorphic in the seven cell lines. We assigned each cell line by genotype to one of three categories, i.e., early-replicating, heterozygous, and late-replicating, at each rtQTL SNP. For each epigenetic feature, we tested whether the cell lines carrying the early-replicating genotypes are more (or less) likely to harbor it than the cell lines carrying the late-replicating genotypes, using the two-tailed binomial test. The binomial parameter *p* was calculated as *p*_late_ × (*p*_perm_early_ / *p*_perm_late_), where *p*_late_ is the proportion of late-replicating genotypes carrying this feature, and *p*_perm_early_ and *p*_perm_late_ are estimated from ten permutations (described below). Bonferroni correction was used to correct for multiple testing at the 0.05 level.

In each permutation, we used random SNPs matched for rtQTLs (for details see the “enrichment analyses” section), and randomly designated one genotype as the early-replicating genotype. We obtained genotype and epigenetic information from the seven cell lines at these random SNPs and calculated the proportion of early- and late-replicating genotypes carrying the feature in ten permutations (*p*_perm_early_ and *p*_perm_late_).

#### Transcription factors

To identify TFs that regulate replication timing, we tested whether rtQTL alleles (in the CAVIAR 90% causal set) influence the binding affinity (motif score) of 21 TFs^77^. Under the hypothesis that some rtQTLs function by altering sequence motifs of TFs that promote or repress replication, early-replicating alleles will be more likely to have higher binding affinities than late-replicating alleles to the TFs that promote earlier replication, and vice versa for late-replicating alleles. We used this principle to identify TFs associated with replication timing. We tested the motifs of all TFs studied in Supplementary Fig. 9a, if available. Of note, SOX2 was not included in this analysis because its motif information was not available. This analysis was repeated with iPSC rtQTLs. We were not able to perform the analysis described above for chromatin states and histone marks with TFs because TF ChIP-seq data was only available for one hESC or iPSC line.

TF binding affinity data, measured by motif score, was obtained from HaploReg^77^. Sequence logos for TF binding motifs were created using WebLogo 3^78^. For each rtQTL SNP, motif scores of the two alleles were obtained for the TFs, and their difference is the log_2_ fold difference in probability that the allele is in a binding motif of the given TF. A higher difference in motif scores means that this SNP can more substantially alter the binding affinity of this TF.

For each TF, we counted the weighted number of rtQTLs for which the early-replicating (or the late-replicating) allele had higher predicted binding affinity, weighted by the difference in motif scores between the two alleles, i.e., rtQTLs with a higher motif score difference will have heavier weight. This weighting scheme assigns heavier weights to those rtQTLs for which the changes in allele state will result in more substantial change in TF binding affinity. If there were more than one potential causal SNPs of an rtQTL located within binding motifs of a given TF, the SNP with the lowest *p* value was used. We compared the numbers to permutations, in which SNPs matched for rtQTLs were randomly selected and the early-replicating alleles were randomly assigned, using the chi-squared test for a 2×2 contingency table. This test assesses whether the early-replicating alleles are more (or less) likely to have higher TF binding affinity than late-replicating alleles than expected by chance. Benjamini-Hochberg correction at 10% FDR was used to correct for multiple testing.

For OCT4, NANOG, and CTCF (for which there are abundant ChIP-seq data available in hESC), we repeated this analysis using only motifs that overlap with TF ChIP-seq peaks (i.e., confirmed TF binding). Consistent with the results in Fig. 5a, we found that OCT4 and NANOG were significantly more likely to bind early-replicating alleles (*p* = 5.97×10^-7^ and 2.62×10^-15^; log_2_ ratio improved from 0.27 and 0.29 to infinity and 2.58, respectively), while CTCF was significantly more likely to bind late-replicating alleles (*p* = 0.02, log_2_ ratio improved from -0.22 to -1.19).

### Reporting summary

Further information on research design is available in the Nature Research Reporting Summary linked to this article.

## Supplementary information

Supplementary Information

Description of Additional Supplementary Files

Supplementary Data 1

Supplementary Data 2

Supplementary Data 3

Supplementary Data 4

Supplementary Data 5

Supplementary Data 6

Supplementary Data 7

Reporting Summary

## Data Availability

All data supporting the findings described in this manuscript are available in the article and in the Supplementary Information, and from the corresponding author upon reasonable request. Due to privacy concerns, genome sequence data of hESC and iPSC lines generated in this study have been desposited in dbGaP under accession number phs001957 and are available under restricted access by application through dbGaP. These data are consented for general research use. PC-corrected replication timing data for individual iPSC cell lines can be accessed at https://www.thekorenlab.org/data. hESC Whole-genome sequence data (from Merkle et al.^19^) are accessible via DUOS (https://duos.broadinstitute.org), listed as dataset DUOS-000121. HipSci data were obtained from https://www.ebi.ac.uk/ena/browser/view/PRJEB15299?show=reads; Roadmap Epigenomics Project data were from http://www.roadmapepigenomics.org/; ENCODE data were from https://www.encodeproject.org/; LCL histone modification data were from Kasowski et al. (ref. ^47^; https://www.ncbi.nlm.nih.gov/geo/query/acc.cgi?acc=GSE50893).

## Code availability

Computer codes used in this study are available from the corresponding author upon request.
