# Supplementary material for: The genetic architecture of DNA replication timing in human pluripotent stem cells
Source: Nat Commun. 2021 Nov 19;12:6746. doi: 10.1038/s41467-021-27115-9 (PMC8604924; doi:10.1038/s41467-021-27115-9)
Supplement: Supplementary file 1 — Supplementary Information [file 41467_2021_27115_MOESM1_ESM.pdf]

Supplementary Information for

The Genetic Architecture of DNA Replication Timing in Human Pluripotent Stem Cells

Qiliang Ding[1], Matthew M. Edwards[1], Ning Wang[2], Alexa N. Bracci[1], Michelle L. Hulke[1], Ya Hu[1,3], Yao Tong[1], Xiang Zhu[4,5,6], Joyce Hsiao[7], Christine J. Charvet[1], Sulagna Ghosh[8,9,10], Robert E. Handsaker[8,9], Kevin Eggan[8,10,11], Florian T. Merkle[12], Jeannine Gerhardt[13,14], Dieter Egli[2], Andrew G. Clark[1], Amnon Koren[1]*.

Correspondence to: koren@cornell.edu

This document includes:

Supplementary Figures 1 to 10
Supplementary Tables 1 to 5

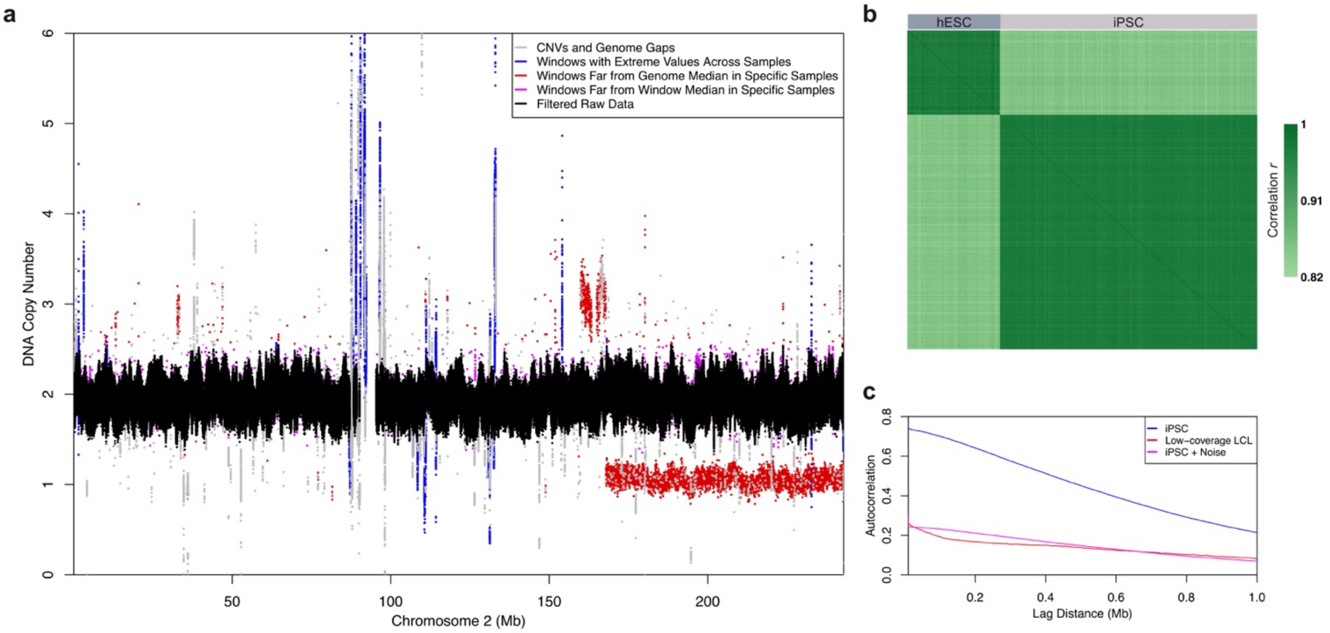

Supplementary Figure 1. Inference and Quality Control of DNA Replication Timing Profiles.

(a) Filtering of outlier regions and data points in the DNA copy number profiles to infer DNA replication timing. Shown are DNA copy number profiles of chromosome 2 for the 108 hESCs. The color of a given data point indicates the first filter through which the data point was removed. The filtering criteria were effective in removing outliers that were likely not driven by replication timing, leaving filtered profiles (black data points) that were optimal for replication timing inference. (b) Correlation matrix of hESC and iPSC replication timing profiles. Correlations were calculated using PC-corrected autosomal replication timing profiles. As expected, hESC and iPSC replication timing profiles are highly similar, with a median correlation of 0.85. (c) Autocorrelation of simulated data with added noise. The low-coverage LCL data (red) used in our earlier work[18] has greater noise than the high-coverage iPSC data (blue) used in the present study, as shown by lower autocorrelation in the plot. Random noise (drawn from a uniform distribution with a = 0 and b = 0.65) was added to the high-coverage iPSC data to simulate an iPSC dataset (magenta) with comparable noise level as the low-coverage LCL data. Autocorrelation is averaged across all cell lines from a given cell type.

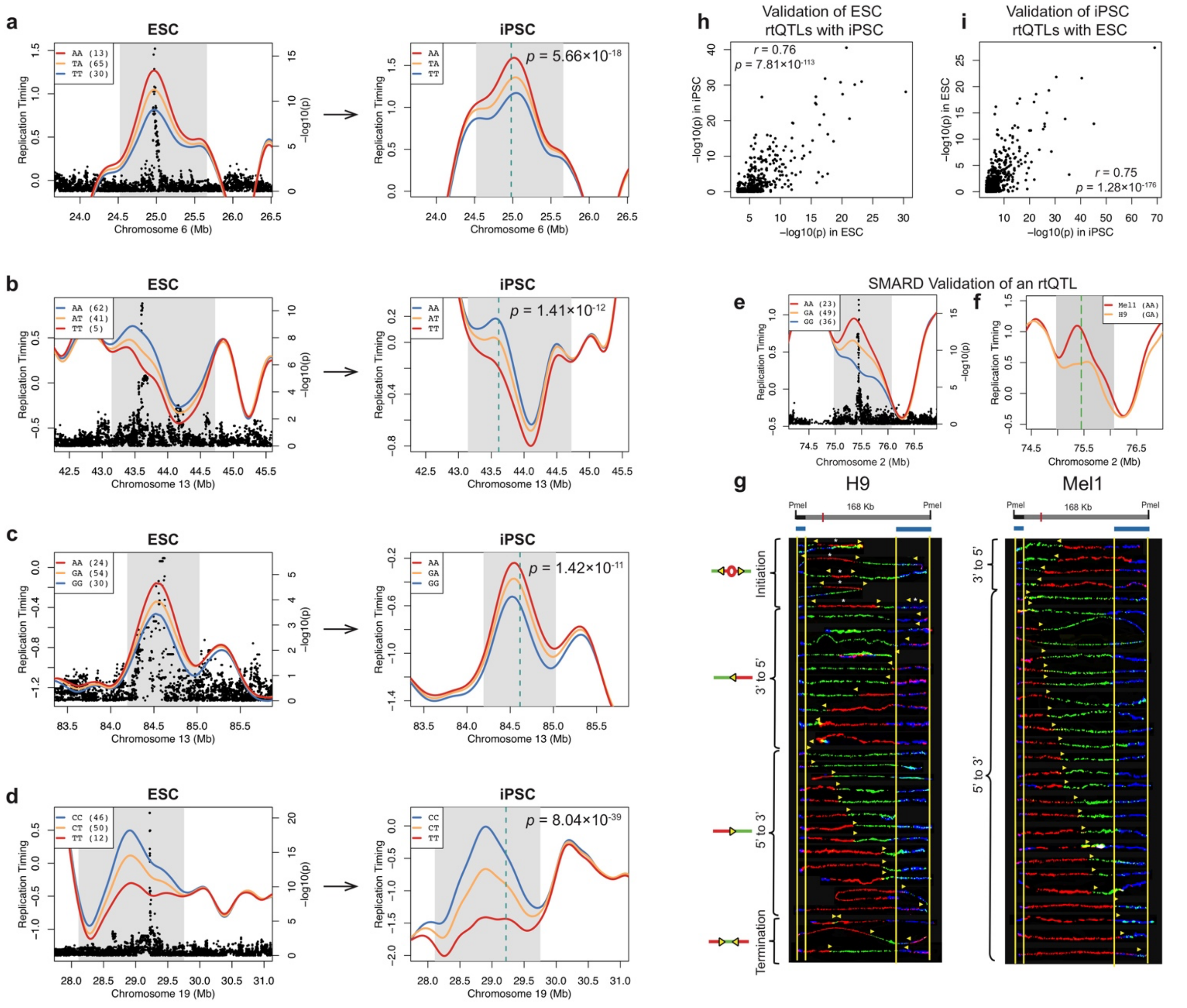

Supplementary Figure 2. rtQTL Validation.

(a–d) Validation of rtQTLs in 192 iPSC lines (Methods; two-sided binomial tests). The left panels are examples of rtQTLs in hESCs. The right panels show replication timing in the same regions in iPSCs, stratified by the genotype of the top rtQTL SNP discovered in the hESCs (vertical line). Association *p*-values in iPSCs are indicated. Excellent agreement between hESCs and iPSCs demonstrate that the rtQTLs discovered in hESCs are reproducible in an independent cohort. (e–g) SMARD (single-molecule analysis of replicated DNA [23]) analysis of an rtQTL on chromosome 2 (Fig. 1i) in Mel1 and H9 cell lines confirms variation in initiation site activity consistent with rtQTL genotypes. (f) Replication timing flanking the rtQTL locus (gray region); green line: the region analyzed by SMARD. The initiation site on the left side of the green line is an rtQTL (panel e), at which Mel1 and H9 carry the early-replicating and heterozygous genotype, respectively. (g) SMARD results, where each line indicates one DNA molecule, and the shift from red to green reveals the location and direction of replication forks (yellow arrows). Significantly more forks are progressing from 5' to 3' in Mel1 when compared with H9 ($p = 4.69\times10^{-4}$, Fisher's exact test, two-sided), indicating that the upstream initiation site is much stronger in Mel1 than H9, consistent with the rtQTL analysis. (h, i) rtQTLs are highly reproducible between the ESCs and iPSCs. When directly testing ESC rtQTLs using iPSCs (h) or *vice versa* (i), the *p*-values show strong positive correlation.

Among the 602 ESC rtQTLs tested, 38.7% (233/602) were validated ($p < 0.05$ and the same direction of effect) in at least one dataset (HipSci iPSC or ESC/iPSC additionally sequenced), much greater than expected ($p = 1.15\times10^{-80}$, binominal test, two-sided). For rtQTLs with $p \leq 5\times10^{-8}$, 85.6% (89/104) were validated ($p = 3.75\times10^{-74}$). Among the iPSC rtQTLs tested, 31.7% (303/955) were validated in ESC ($p << 2.2\times10^{-16}$). For iPSC rtQTLs with $p \leq 5\times10^{-8}$, 82.3% (149/181) were validated ($p << 2.2\times10^{-16}$).

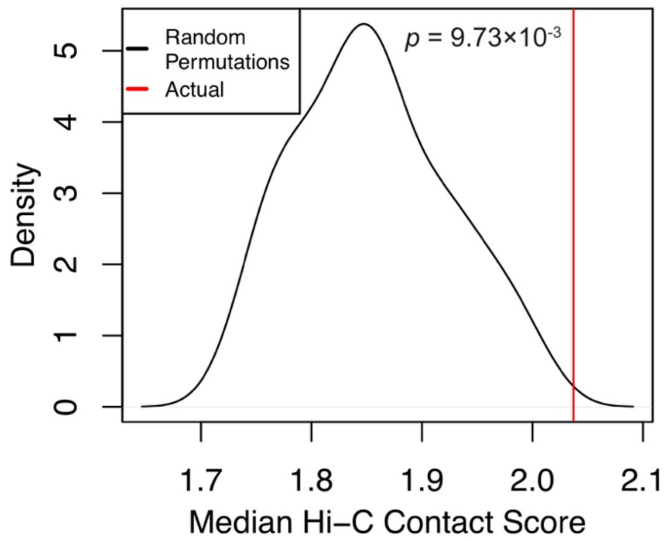

Supplementary Figure 3. Primary and Secondary rtQTLs have Significantly More 3D Contacts than Expected by Chance.

The median Hi-C contact score for all primary-secondary rtQTL pairs was 2.037 (red vertical bar). This value is significantly higher than 100 random permutations (black distribution), in which the distances between primary and secondary rtQTLs were preserved but actual genomic locations were randomly shifted. We confirmed the normality assumption of the plotted distribution using the Wilks-Shapiro test ($p > 0.05$; two-sided).

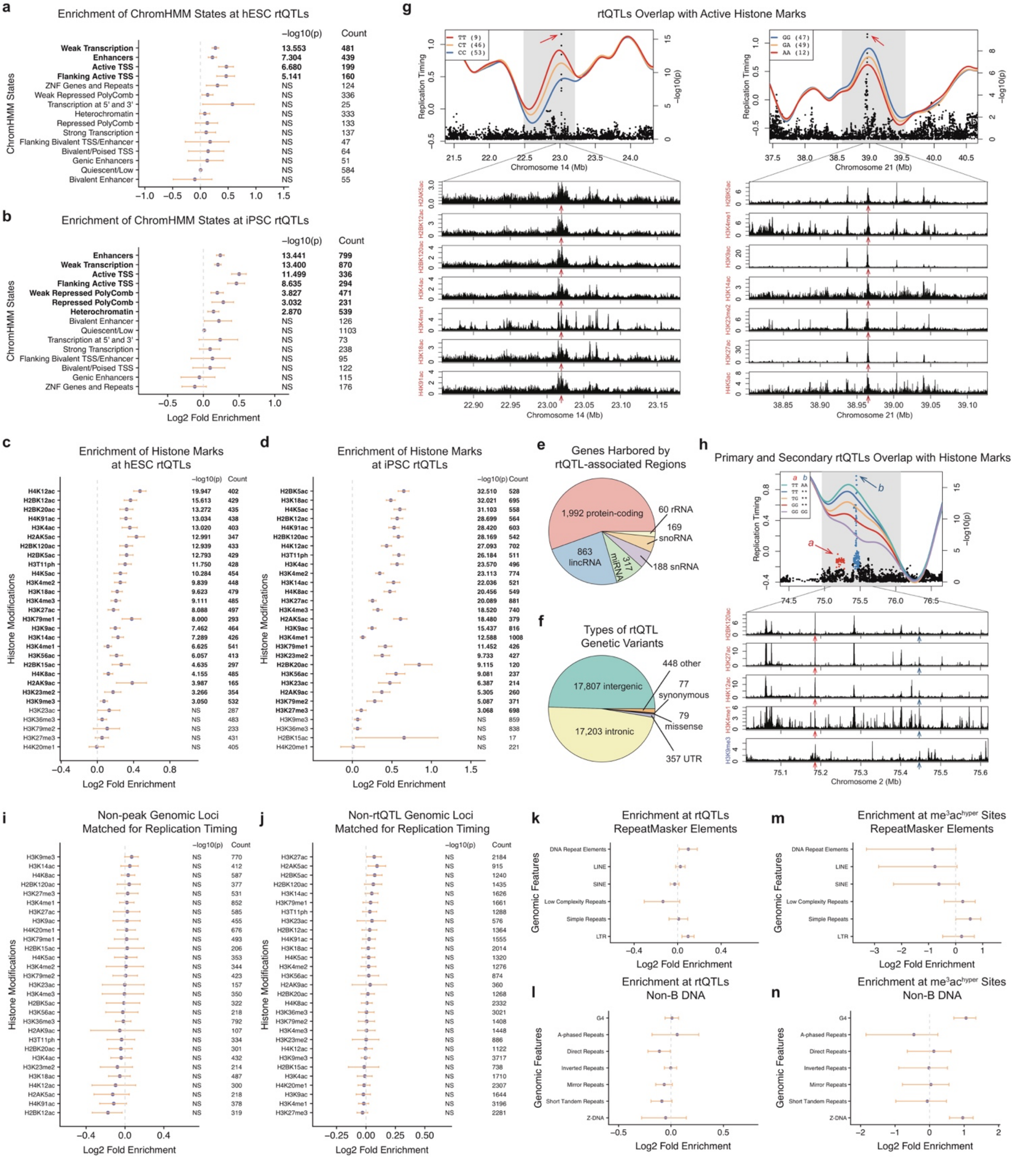

Supplementary Figure 4. rtQTLs are Enriched for Active Chromatin States and Histone Marks.

(a, b) Enrichment of chromHMM chromatin states at rtQTLs identified in hESCs (a) or iPSCs (b). Orange bars: 95% confidence intervals. NS: not significant at Bonferroni-corrected $p = 0.05$. (c, d) Enrichment of histone marks at hESC (c) and iPSC (d) rtQTLs. Similar to panels a and b. (e) Breakdown of gene types located within rtQTL-associated regions. The number of genes in rtQTL-associated regions was significantly lower than expected (p =

4.85×10^{-17}, two-sided *Z*-test) without enrichment for gene ontology terms[44]. (f) Functional annotations of rtQTL genetic variants. (g) rtQTLs colocalize with active histone modifications. Bottom panels: hESC ChIP-seq tracks of active histone modifications. Imputed histone tracks[45,46] from the Roadmap Epigenomics Project were used. Red arrows: locations of rtQTL variants indicated in the top panels. (h) A multi-rtQTL region (same as Fig. 2c) at which both the primary and secondary rtQTLs overlap active histone marks. (i, j) No enrichment in histone marks was observed at non-peak (i) and non-rtQTL (j) loci that match the replication timing of peaks or rtQTLs, respectively. hESC replication timing profiles were used, with the same enrichment analysis procedures as used for rtQTLs (panel c). (k–n) Enrichment analysis of RepeatMasker repetitive elements (k, m) and non-B DNA motifs (l, n) at rtQTLs (k, l) and the histone modification sites (m, n). Orange bars: 95% confidence intervals. LINE: long interspersed nuclear elements. SINE: short interspersed nuclear elements. LTR: long terminal repeats. Significant enrichments were only observed for G4 and Z-DNA motifs at the histone modification sites (panel n). n = 592 genomic regions analyzed in panels a, c, k, l, m, n; n = 1,126 genomic regions analyzed in panels b and d; n = 2,663 genomic regions analyzed in panel i; n = 12,003 genomic regions analyzed in panel j. All error bars are 95% confidence intervals. For panels a, b, c, d, i, j, k, l, m, and n, the center of the error bars in median. These panels used the Binomial test; the displayed -log10(p-values) were uncorrected, but the significance threshold was corrected for multiple testing.

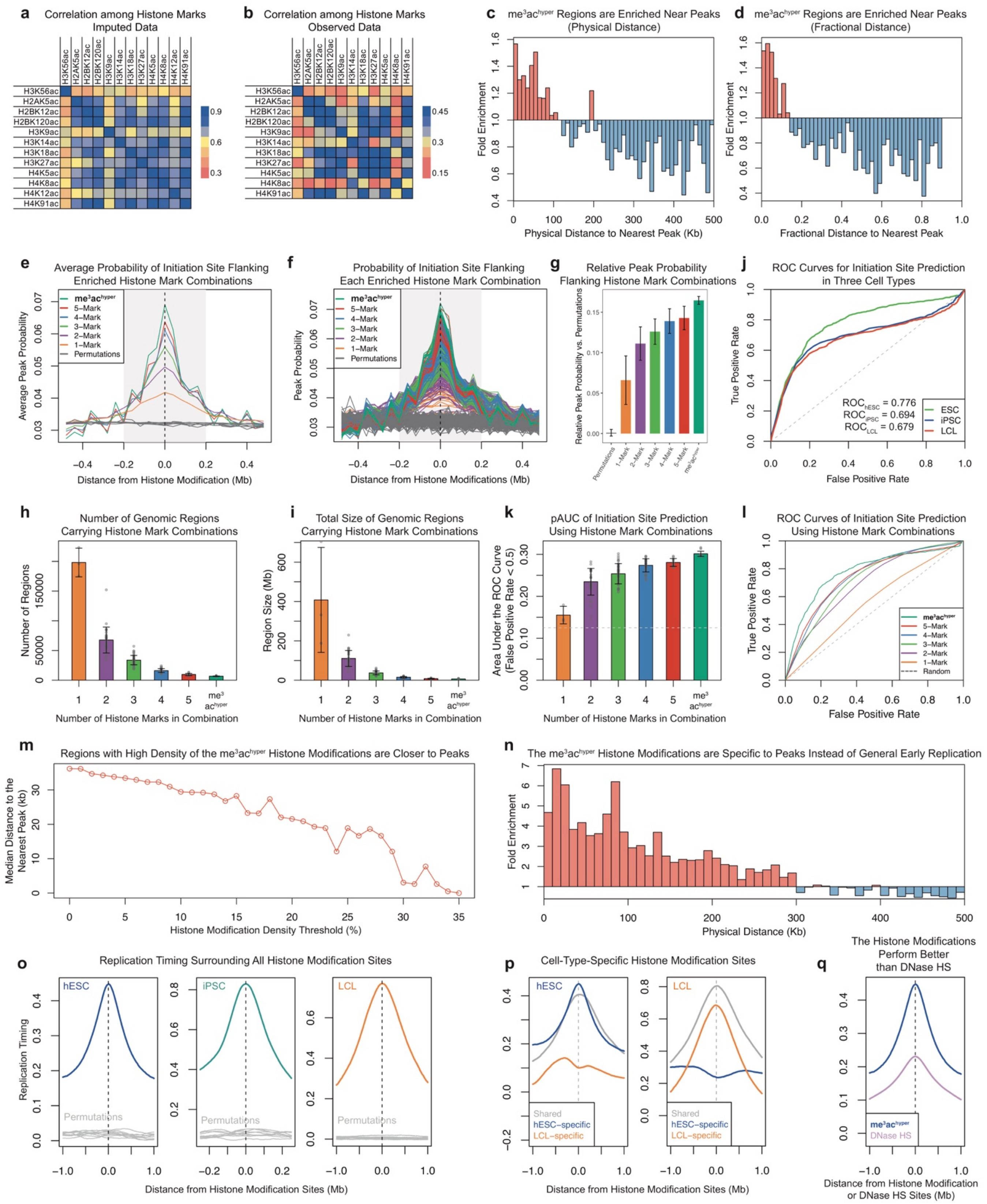

Supplementary Figure 5. Further Support for Histone Modification Combinations for Human DNA Replication Initiation Sites.

(a, b) Correlation of histone acetylation marks at hESC rtQTL sites, using imputed (a) or observed (b) data. (c, d) me^3ac^{hyper} regions are closer to peaks than permutations. Bins containing <=3 regions were excluded. (e) Histone mark combinations correspond to replication initiation sites. (f) Probability of having an initiation site as a function of distance from histone marks (40 kb bins), for each individual histone combination. (g) Normalized cumulative probability of initiation sites within 200 kb of individual histone marks/combinations. Probabilities were normalized by subtracting the permutation mean. Error bars: standard deviation. n = 414 permutations (first bar), n = 27, 160, 129, 72, 13, 13 histone modifications or combinations (for 1/2/3/4/5-mark and me^3ac^{hyper}, respectively). (h, i) Number (h) and total size (i) of regions of histone marks or combinations. Error bars: standard deviation. (j) ROC curves for histone modifications predicting replication initiation sites. Diagonal lines: random guesses. (k, l) ROC analysis of histone marks and combinations. (k) Distribution of pAUC for histone marks and combinations. Partial area under the left half of the curve was calculated. Error bars: standard deviation. Horizontal bar: pAUC of random guess. In panels h, i, and k: n = 3, 29, 48, 34, 12, 13 histone modifications or combinations (for 1/2/3/4/5-mark and me^3ac^{hyper}, respectively). In panels g, h, i, and k, the bars represent means. (l) Averaged ROC curves for histone marks or combinations. (m) Genomic regions with high density of the me^3ac^{hyper} histone modifications are closer to replication timing peaks than isolated histone modification regions. (n) Histone modifications are specific to replication timing peaks. Comparison between distance from histone modification regions to actual replication timing peaks and regions with similar replication timing (distant from peaks). (o) Cumulative replication timing surrounding histone modifications. Gray: ten permutations. (p) Cumulative replication timing in hESCs and LCLs surrounding histone modification locations found in both cell types (gray), LCLs only (orange), or hESCs only (blue). (q) Histone modification combinations are more specific at predicting replication timing peaks than DNase hypersensitivity sites.

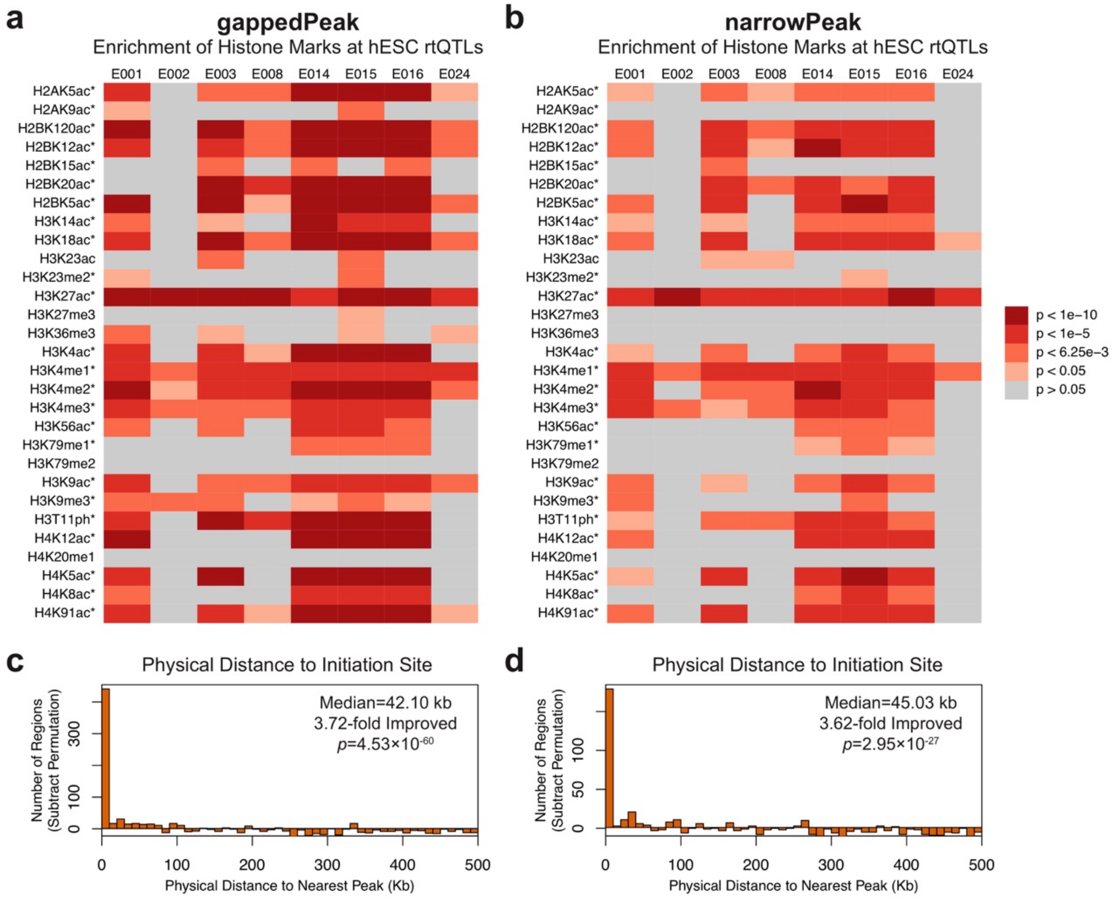

Supplementary Figure 6. Validation of Histone Enrichments in Individual Cell Lines and Using "Gapped" or "Narrow" Peak.

(a, b) Enrichment of histone marks at hESC rtQTLs, using gappedPeak (panel a) or narrowPeak (panel b) data from individual hESC lines. Each column represents an hESC line and each row represents a histone mark. The E001, E002, E003, E008, E014, E015, E016, E024 cell lines are also known as I3, H7, H1, H9, HUES48, HUES6, HUES64, and UCSF4, respectively. Asterisk: also enriched using the union of histone mark data from all eight hESC lines (Fig. S4c). In panel a, all histone marks with an asterisk were enriched in at least one hESC line ($p < 6.25\times10^{-3}$, i.e., Bonferroni-corrected for the 8 hESC lines), while 20 of them were enriched in at least four hESC lines. In panel b, 22 of the 24 histone marks with an asterisk were enriched in at least one hESC line. Panels a and b used the Binominal test; the displayed -log10(p-values) were uncorrected for multiple testing, Bonferroni correction was used when determining significance threshold. (c, d) me^3ac^{hyper} regions identified using gappedPeak (panel c) or narrowPeak (panel d) data from individual hESC lines both show close proximity to replication initiation sites. There were a total of 5,120 and 2,252 me^3ac^{hyper} regions identified across eight hESC lines using gappedPeak or narrowPeak data, respectively. These results reproduce those in Fig. S4c and Fig. 3e using data from individual hESC lines as well as using narrowPeak data. For panels c and d, a One-sided Wilcoxon rank-sum test was used; correction for multiple testing was not applicable as there was only one test for each sub-plot.

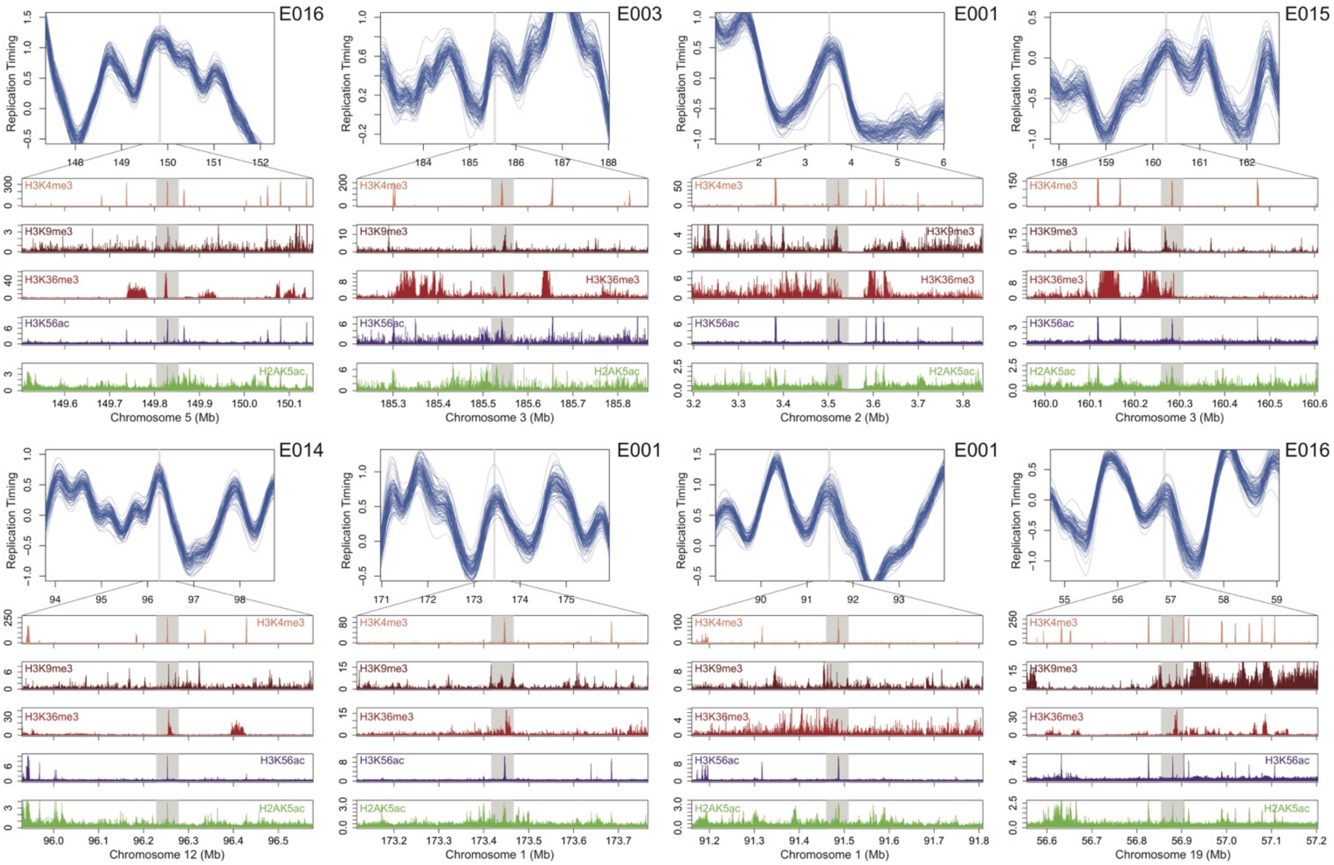

Supplementary Figure 7. Examples of me^3ac^{hyper} Regions in Individual hESC Lines.

Similar to Fig. 3d. Roadmap Epigenomics Project data was used for histone signal track plotting. Experimentally measured data (i.e., “observed”) data was used whenever available, and computationally imputed data was used otherwise. For simplicity, only one of the variable acetylation marks is plotted. The specific hESC line from which the histone signal tracks were derived is indicated in each panel.

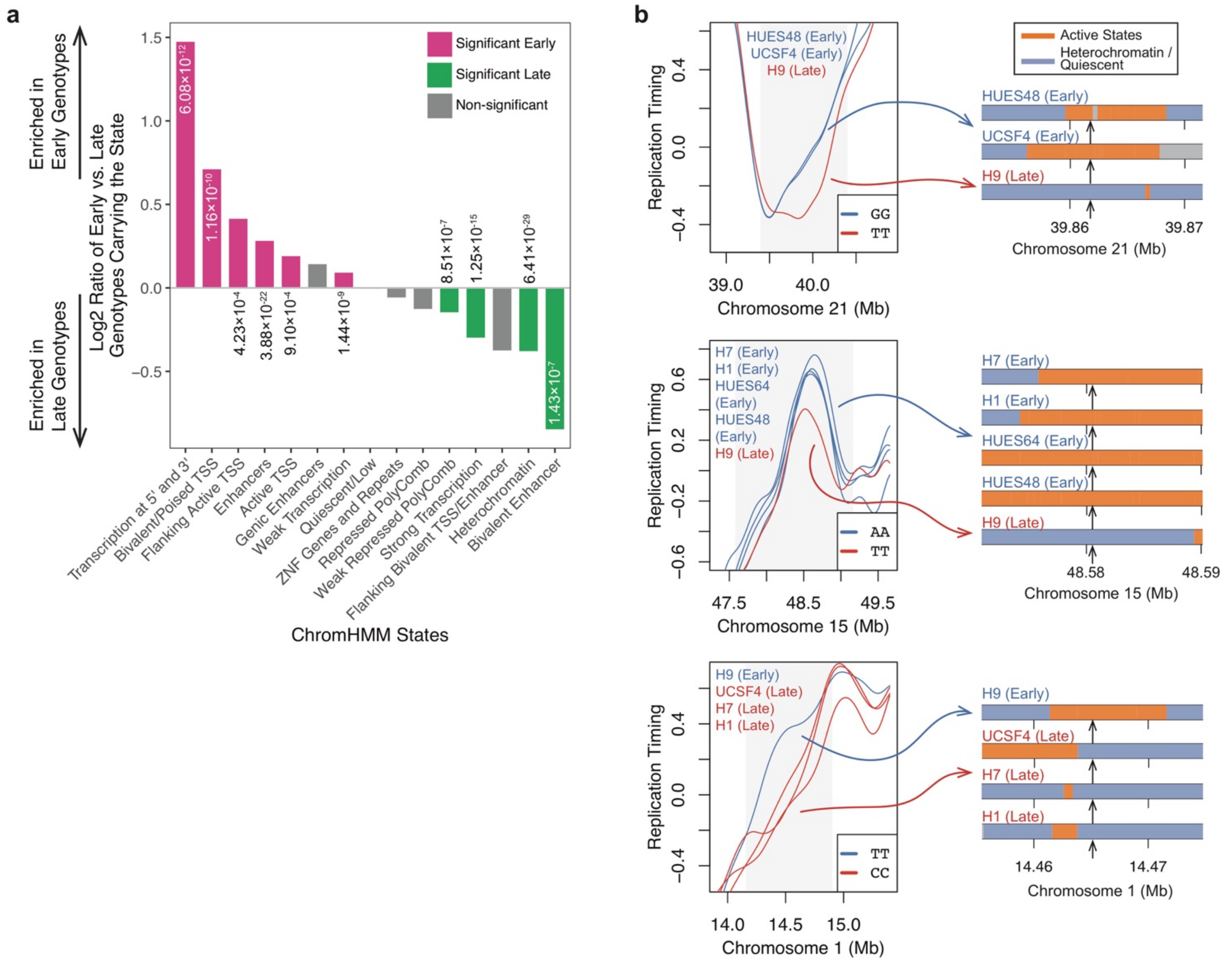

Supplementary Figure 8. rtQTLs Impact Replication Timing by Affecting Chromatin States.

(a) Associations of rtQTL genotypes with chromHMM states. Positive values indicate that the early-replicating genotypes are more likely to carry a given chromatin state, and *vice versa* for negative / late genotypes. (b) Examples of rtQTLs associated with chromatin states. The right panels show chromatin states flanking the rtQTL in the same cell lines. Orange: active states (TSS, enhancer, or weak transcription), blue: heterochromatin or quiescent states, gray: other states.

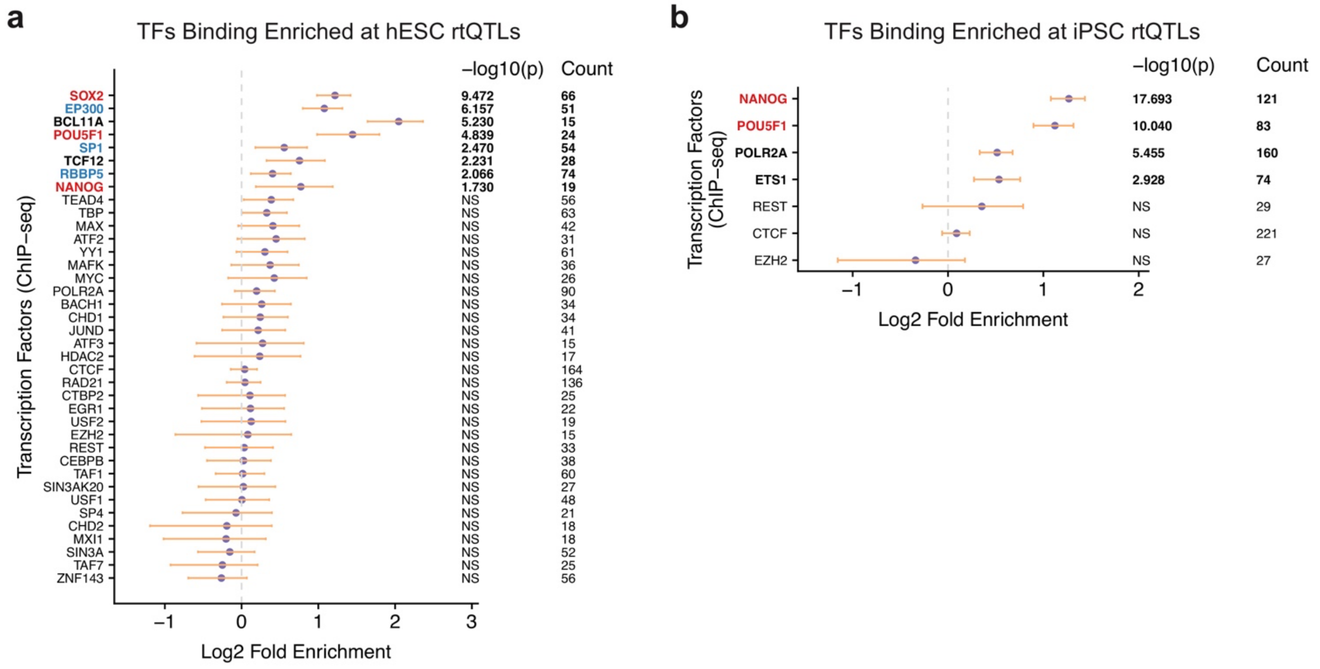

Supplementary Figure 9. Enrichment of TFs at hESC (a) and iPSC (b) rtQTLs.

rtQTLs are enriched at binding sites of central pluripotency factors (red) and chromatin remodelers (blue). NS: not significant at 10% FDR. Only TFs overlapping with at least 15 rtQTLs are plotted. (a) n = 592 genomic regions; (b) n = 1,126 genomic regions; All error bars are 95% confidence intervals.

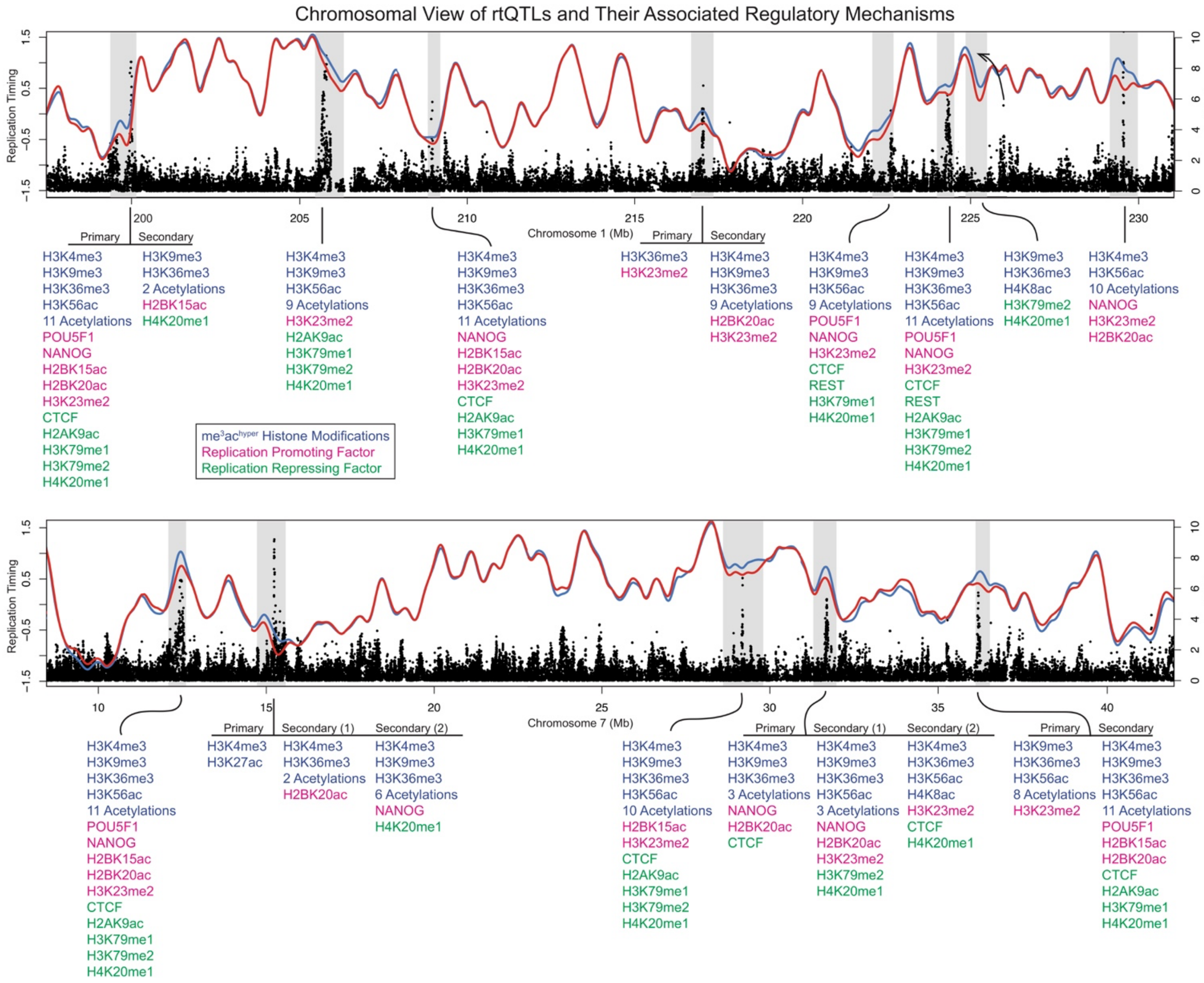

Supplementary Figure 10. rtQTLs Regulate Replication Timing via Numerous Activating and Repressing Effectors.

Different combinations of TFs and histone marks exert positive and negative effects on subsets of replication initiation sites. Both examples show 10 ESC rtQTLs spanning a ~30-Mb region (on chromosomes 1 and 7). The blue and red lines are mean replication profiles of individuals carrying the early- and late-replicating genotypes, respectively. The rtQTL at 225 Mb of chromosome 1 exerts a long-range effect (arrow). Histone marks and TFs overlapping rtQTL genetic variants are shown below. They include positive (magenta) and negative (green) determinants of replication timing (Fig. 4 and 5), and instances of the replication initiation histone modifications (blue, Fig. 3).

Supplementary Table 1. Overlap of rtQTL Genetic Variants with the Enriched 5-mark Histone Mark Combinations

Histone Mark Combination	Number of rtQTLs	Fold-enrichment	*P*-value
H3K4me3-H3K9me3-H3K36me3-H3K56ac-H3K14ac	56	3.71	3.02×10^{-55}
H3K4me3-H3K9me3-H3K36me3-H3K56ac-H3K18ac	62	3.65	4.59×10^{-56}
H3K4me3-H3K9me3-H3K36me3-H3K56ac-H3K27ac	60	3.72	1.81×10^{-57}
H3K4me3-H3K9me3-H3K36me3-H3K56ac--H2BK12ac	49	3.71	1.62×10^{-56}
H3K4me3-H3K9me3-H3K36me3-H3K56ac-H3K4me1	57	3.42	1.58×10^{-45}
H3K4me3-H3K9me3-H3K36me3-H3K56ac-H2AK5ac	44	3.75	1.18×10^{-51}
H3K9me3-H3K36me3-H3K56ac-H2AK5ac-H4K12ac	45	3.56	1.72×10^{-52}
H3K4me3-H3K9me3-H3K36me3-H3K56ac-H2BK120ac	51	3.68	2.00×10^{-56}
H3K4me3-H3K9me3-H3K36me3-H3K56ac-H4K5ac	57	3.73	2.61×10^{-57}
H3K4me3-H3K9me3-H3K36me3-H3K56ac-H4K91ac	56	3.80	5.67×10^{-59}
H3K4me3-H3K9me3-H3K36me3-H4K8ac-H3K27ac	69	3.44	4.62×10^{-70}
H3K4me3-H3K9me3-H3K36me3-H3K56ac-H4K8ac	60	3.57	1.21×10^{-54}
H3K4me3-H3K9me3-H3K36me3-H3K9ac-H4K8ac	71	2.98	8.98×10^{-50}
Any of the 5-mark combinations (union)	89	2.63	3.80×10^{-67}

Supplementary Table 2. Details of Statistical Tests and Estimates

Name	Name of the Test or Estimate	*P*-value or Estimate	Uncertainty Measure
SMARD, number of 5' to 3' forks	Fisher's exact test	4.69×10^{-4}	95% CI: 2.15 to 41.04
Siblings vs. unrelated samples, correlation	Wilcoxon rank-sum test	2.45×10^{-6}	W = 107390
IBD sharing, correlation	ANOVA	3.81×10^{-4}	F = 12.62, df = 1, 22126
Same vs. different donors, correlation	Wilcoxon rank-sum test	8.17×10^{-23}	W = 3728250
Validation of hESC rtQTLs in at least one dataset	Binomial test	1.15×10^{-80}	95% CI: 0.35 to 0.43
Validation of hESC rtQTLs in at least one dataset, $p < 5\times10^{-8}$	Binomial test	3.75×10^{-74}	95% CI: 0.77 to 0.92
Validation of iPSC rtQTLs in hESC	Binomial test	$<< 2.2\times10^{-16}$	95% CI: 0.29 to 0.35
Validation of iPSC rtQTLs in hESC, $p < 5\times10^{-8}$	Binomial test	$<< 2.2\times10^{-16}$	95% CI: 0.76 to 0.88
Primary rtQTLs closer to peak than secondary rtQTLs	Wilcoxon rank-sum test	3.28×10^{-8}	W = 17176
Primary and secondary rtQTLs cluster in space (Hi-C)	Z-test	9.73×10^{-3}	Z = 2.59
Number of early-replicating alleles vs. replication timing, regions w/ 2 rtQTLs	Linear regression	$<< 2.2\times10^{-16}$	95% CI: 0.32 to 0.33
Number of early-replicating alleles vs. replication timing, regions w/ 3 rtQTLs	Linear regression	$<< 2.2\times10^{-16}$	95% CI: 0.27 to 0.30
Correlation of replication timing among samples	Median	0.93	95% range: 0.81 to 0.97
Number of fine-mapped SNPs per rtQTL	Median	33	95% range: 5 to 213
Size of me^3ac^{hyper} histone modification regions	Median	635 bp	95% range: 81 to 2412 bp
Inter-origin distance	Median	971.2 kb	95% range: 228 to 2293 kb
Size of regions influenced by rtQTLs	Average	858 kb	95% range: 406 to 1647 kb
Number of histone marks overlapped with rtQTLs	Average	20	95% range: 4 to 29

Supplementary Table 3. Results of rtQTL Mapping are Highly Robust to the Thresholds Chosen, Part 1.

a	*b*	Proportion of Genome Associated with rtQTLs	Average Size of rtQTL-associated Regions (kb)	Mean Number of rtQTLs at Multi-rtQTL Regions
0.1	0.1	0.127	819.19	2.24
0.1	0.2	0.128	818.77	2.24
0.1	0.5	0.133	827.26	2.35
0.1	0.8	0.135	832.83	2.44
0.2	0.1	0.136	807.28	2.52
0.2	0.2	0.136	806.56	2.53
0.2	0.5	0.137	815.89	2.58
0.2	0.8	0.138	824.22	2.66
0.5	0.1	0.136	792.43	2.81
0.5	0.2	0.136	792.43	2.81
0.5	0.5	0.137	802.11	2.86
0.5	0.8	0.138	813.82	2.95
0.8	0.1	0.136	793.41	2.81
0.8	0.2	0.136	793.41	2.81
0.8	0.5	0.137	803.08	2.86
0.8	0.8	0.138	814.75	2.97

Supplementary Table 4. Results of rtQTL Mapping are Highly Robust to the Thresholds Chosen, Part 2.

c (Mb)	Proportion of Genome Associated with rtQTLs	Average Size of rtQTL-associated Regions (kb)	Mean Number of rtQTLs at Multi-rtQTL Regions
0.5	0.136	814.08	2.57
1	0.136	806.32	2.53
2	0.136	807.28	2.52
3	0.136	807.28	2.52
4	0.136	807.28	2.52
5	0.136	807.28	2.52

Supplementary Table 5. Multi-rtQTL Results are Robust to the Distance Threshold Chosen.

d (Mb)	Total Multi-rtQTL Regions	Mean Number of rtQTLs at Multi-rtQTL Regions	Max Number of rtQTLs at Multi-rtQTL Regions
0.25	118	2.31	5
0.5	127	2.45	5
1	134	2.48	6
2	135	2.52	6
3	139	2.59	7
5	141	2.68	7
10	150	2.83	8
