# Supplementary material for: The genetic architecture of DNA replication timing in human pluripotent stem cells
Source: Nat Commun. 2021 Nov 19;12:6746. doi: 10.1038/s41467-021-27115-9 (PMC8604924; doi:10.1038/s41467-021-27115-9)
Supplement: Supplementary file 2 — Description of Additional Supplementary Files [file 41467_2021_27115_MOESM2_ESM.pdf]

Description of Additional Supplementary Files

File Name: Supplementary Data 1
Description: List of rtQTLs Identified in This Study. The fastQTL package was used to calculate p-values for rtQTLs. The presented p-values were uncorrected for multiple testing. Correction for multiple testing was used when identifying significant associations (see Methods for details).

File Name: Supplementary Data 2
Description: Fine-mapped Genetic Variants of hESC rtQTLs by CAVIAR.

File Name: Supplementary Data 3
Description: Histone modifications associated with each hESC rtQTL.

File Name: Supplementary Data 4
Description: Locations of Histone modification Sites in hESCs.

File Name: Supplementary Data 5
Description: Locations of Replication Timing Peaks in hESCs.

File Name: Supplementary Data 6
Description: Locations of Histone modification Sites in iPSCs.

File Name: Supplementary Data 7
Description: Locations of Replication Timing Peaks in iPSCs.
