# Supplementary material for: The genetic architecture of DNA replication timing in human pluripotent stem cells
Source: Nat Commun. 2021 Nov 19;12:6746. doi: 10.1038/s41467-021-27115-9 (PMC8604924; doi:10.1038/s41467-021-27115-9)
Supplement: Supplementary file 10 — Reporting Summary [file 41467_2021_27115_MOESM10_ESM.pdf]

nature research

Corresponding author(s): Amnon Koren

updated by author(s): October 18, 2021

Reporting Summary

Nature Research wishes to improve the reproducibility of the work that we publish. This form provides structure for consistency and transparency in reporting. For further information on Nature Research policies, see our Editorial Policies and the Editorial Policy Checklist.

Statistics

For all statistical analyses, confirm that the following items are present in the figure legend, table legend, main text, or Methods section.

n/a	Confirmed	
☐	☒	The exact sample size (*n*) for each experimental group/condition, given as a discrete number and unit of measurement
☒	☐	A statement on whether measurements were taken from distinct samples or whether the same sample was measured repeatedly
☐	☒	The statistical test(s) used AND whether they are one- or two-sided<br>*Only common tests should be described solely by name; describe more complex techniques in the Methods section.*
☐	☒	A description of all covariates tested
☐	☒	A description of any assumptions or corrections, such as tests of normality and adjustment for multiple comparisons
☐	☒	A full description of the statistical parameters including central tendency (e.g. means) or other basic estimates (e.g. regression coefficient) AND variation (e.g. standard deviation) or associated estimates of uncertainty (e.g. confidence intervals)
☐	☒	For null hypothesis testing, the test statistic (e.g. *F*, *t*, *r*) with confidence intervals, effect sizes, degrees of freedom and *P* value noted<br>*Give P values as exact values whenever suitable.*
☒	☐	For Bayesian analysis, information on the choice of priors and Markov chain Monte Carlo settings
☒	☐	For hierarchical and complex designs, identification of the appropriate level for tests and full reporting of outcomes
☐	☒	Estimates of effect sizes (e.g. Cohen's *d*, Pearson's *r*), indicating how they were calculated

Our web collection on statistics for biologists contains articles on many of the points above.

Software and code

Policy information about availability of computer code

Data collection	GenomeSTRiP v2.00
Data analysis	Perl 5, R (3.5.0, 3.6.1), fastQTL v2.0, TRUFFLE v1.38, CAVIAR v2.0, MATLAB, IMPUTE2 v2.3.2, bwa v0.7.13, Picard Tools (v2.9.0), GATK

For manuscripts utilizing custom algorithms or software that are central to the research but not yet described in published literature, software must be made available to editors and reviewers. We strongly encourage code deposition in a community repository (e.g. GitHub). See the Nature Research guidelines for submitting code & software for further information.

Data

Policy information about availability of data

All manuscripts must include a data availability statement. This statement should provide the following information, where applicable:

- Accession codes, unique identifiers, or web links for publicly available datasets
- A list of figures that have associated raw data
- A description of any restrictions on data availability

Data of hESC and iPSC lines sequenced in this study were desposited in dbGaP (accession number: phs001957). (https://www.ncbi.nlm.nih.gov/projects/gapprev/gap/cgi-bin/study.cgi?study_id=phs001957.v1.p1) and are available under restricted access.

April 2020

Field-specific reporting

Please select the one below that is the best fit for your research. If you are not sure, read the appropriate sections before making your selection.

☒ Life sciences ☐ Behavioural & social sciences ☐ Ecological, evolutionary & environmental sciences

For a reference copy of the document with all sections, see nature.com/documents/nr-reporting-summary-flat.pdf

Life sciences study design

All studies must disclose on these points even when the disclosure is negative.

Sample size	No statistical method was used to predetermine sample size. To maximize statistical power, we used all cell lines with available replication timing information in rtQTL mapping, except for samples used for validation. Our sample sizes are appropriate because they are comparable with human eQTL mapping studies.
Data exclusions	For rtQTL mapping in hESCs, we removed eight samples because of non-European ancestry. This exclusion criterion was pre-established.
Replication	We mapped rtQTLs in two similar cell types - hESCs and iPSCs. We successfully replicated rtQTLs between these two datasets. Furthermore, we successfully replicated hESC rtQTLs using three additional datasets (totaling 17 iPSCs and 32 hESCs). A locus-specific single-molecule assay was used also to validate an rtQTL in hESC.
Randomization	This is not relevant because our study did not require allocation of samples to experimental groups.
Blinding	This is not relevant because this is a cohort study.

Reporting for specific materials, systems and methods

We require information from authors about some types of materials, experimental systems and methods used in many studies. Here, indicate whether each material, system or method listed is relevant to your study. If you are not sure if a list item applies to your research, read the appropriate section before selecting a response.

Materials & experimental systems

n/a	Involved in the study	
☒	☐	Antibodies
☐	☒	Eukaryotic cell lines
☒	☐	Palaeontology and archaeology
☒	☐	Animals and other organisms
☒	☐	Human research participants
☒	☐	Clinical data
☒	☐	Dual use research of concern

Methods

n/a	Involved in the study	
☒	☐	ChIP-seq
☒	☐	Flow cytometry
☒	☐	MRI-based neuroimaging

Eukaryotic cell lines

Policy information about cell lines

Cell line source(s)	CHB1, CHB2, CHB3 (Lerou et al. 2008); CuES1, CuES2, CuES3, CuES6, MR14, MR15, MR16, MR18, MR20, 1016B, 1078, 1106-4, 1128A, 1135B, 1141-1, 1224B, 1225A, C9012, FA0003 (unpublished); CuES4 (Sagi et al. 2019); HuES48 (Chen et al. 2009); HuES6 (Cowan et al. 2004); 1018A, 1018E, BJiPSM, BJiPSO (Johannesson et al. 2014, Sui et al. 2017); 1158F, 1159B (Sui et al. 2017); C2A (Si-Tayeb et al. 2010).
Authentication	All cell lines have been authenticated by whole-genome sequencing and SNP genotyping.
Mycoplasma contamination	All cell lines tested negative for mycoplasma.
Commonly misidentified lines (See ICLAC register)	No commonly misidentified lines (version 10, Mar 25, 2020) were used in this study.
